# Novel Mucosal DNA-MVA HIV Vaccination in Which DNA-IL-12 Plus Cholera Toxin B Subunit (CTB) Cooperates to Enhance Cellular Systemic and Mucosal Genital Tract Immunity

**DOI:** 10.1371/journal.pone.0107524

**Published:** 2014-09-12

**Authors:** Cynthia Maeto, Ana María Rodríguez, María Pía Holgado, Juliana Falivene, María Magdalena Gherardi

**Affiliations:** Instituto de Investigaciones Biomédicas en Retrovirus y SIDA (INBIRS), Universidad de Buenos Aires- CONICET, Buenos Aires, Argentina; University of Cape Town, South Africa

## Abstract

Induction of local antiviral immune responses at the mucosal portal surfaces where HIV-1 and other viral pathogens are usually first encountered remains a primary goal for most vaccines against mucosally acquired viral infections. Exploring mucosal immunization regimes in order to find optimal vector combinations and also appropriate mucosal adjuvants in the HIV vaccine development is decisive. In this study we analyzed the interaction of DNA-IL-12 and cholera toxin B subunit (CTB) after their mucosal administration in DNA prime/MVA boost intranasal regimes, defining the cooperation of both adjuvants to enhance immune responses against the HIV-1 Env antigen. Our results demonstrated that nasal mucosal DNA/MVA immunization schemes can be effectively improved by the co-delivery of DNA-IL-12 plus CTB inducing elevated HIV-specific CD8 responses in spleen and more importantly in genital tract and genito-rectal draining lymph nodes. Remarkably, these CTL responses were of superior quality showing higher avidity, polyfunctionality and a broader cytokine profile. After IL-12+CTB co-delivery, the cellular responses induced showed an enhanced breadth recognizing with higher efficiency Env peptides from different subtypes. Even more, an *in vivo* CTL cytolytic assay demonstrated the higher specific CD8 T-cell performance after the IL-12+CTB immunization showing in an indirect manner its potential protective capacity. Improvements observed were maintained during the memory phase where we found higher proportions of specific central memory and T memory stem-like cells T-cell subpopulations. Together, our data show that DNA-IL-12 plus CTB can be effectively employed acting as mucosal adjuvants during DNA prime/MVA boost intranasal vaccinations, enhancing magnitude and quality of HIV-specific systemic and mucosal immune responses.

## Introduction

Natural transmission of HIV and SIV occurs predominantly via mucosal surfaces, which are the major entry points of these viruses and concomitantly are the first line of host defense to combat the infection. Once the mucosal epithelial barrier is crossed, a small founder population of infected cells is rapidly established. Then, local viral expansion occurs during the first week and later, a self-propagating systemic infection throughout the secondary lymphoid organs is established [1], [2]. Thus, the small infected founder populations implied during HIV-1 mucosal transmission clearly indicate that the greatest opportunities for prevention may be strategies that target these initially small and genetically homogeneous foci of mucosal infection in the first week of infection [2]. However, despite evidences related to the kinetic characteristics of the infection and the mucosal natural transmission of the virus, mucosal surfaces are not targeted by most HIV vaccines currently under trial (http://www.iavi.org). Conversely, most of the research emphasis is focused on the analysis of systemic routes of inoculation, mainly the intramuscular one.

The stimulation of the mucosal immune response can be achieved by the administration of immunogens at mucosal inductive sites, where specialized organized lymphoepithelial follicular structures exist. The concept of a common mucosa- associated system regulating and coordinating immune response at mucosal surfaces implied an important advance in our understanding of protection against mucosal pathogens. This system, called the mucosa-associated lymphoid tissue, is based on primed T and B lymphocytes that migrate from the site of antigen presentation via the lymphatic and blood to selectively home to lymphoid tissue at distant sites in gastrointestinal, respiratory, genitourinary, and other mucosa-associated regions [3]. Various studies have demonstrated that both oral and intranasal administration of antigens are capable of inducing immune responses at distant effector sites [4]. In this sense, the use of the intranasal route to stimulate inductive sites in the respiratory tract has been of considerable interest over the past years, demonstrating to be a feasible mucosal route to effectively induce both systemic and mucosal immune responses at distal places after mice [5], monkeys [6], [7] as well as humans immunization [8].

Evidences from experimental data and from natural HIV infection suggest that in order to control both entry and systemic dissemination, an effective HIV vaccine may need to stimulate both arms of the adaptive immune system, eliciting cellular and humoral immunity systemically as well as at mucosal surfaces.

The application of DNA/MVA vaccine vectors expressing HIV antigens by mucosal routes have been previously explored in macaques [9] and diverse mice models [10]. In a previous study, we were able to demonstrate that a MVA vector expressing HIV gp160 intranasally delivered after MVAenv/MVAenv and DNAenv/MVAenv schemes, plus the experimental mucosal adjuvant cholera toxin (CT), induced mucosal CD8 T- cell responses and also local antibodies in the genital tract and genito-rectal draining lymph nodes [5]. Although CT is the mucosal adjuvant most employed for research experiments, its application is not possible in human trials due to its toxicity. Thus, the identification and characterization of adjuvant-molecules is very important for mucosal immunization approaches in order to induce long lasting protective immunity. On the other hand, to the extent in which vaccines need to be focused not only on creating an immune response against an antigen, but also on the quality of that response, the role of adjuvants is becoming increasingly significant. Even more, over the past years several evidences indicated that the main characteristic that is really relevant in anti-viral T-cell immunity is its quality in terms of the type of cytokines secreted, cytotoxic activity, proliferative capacity, etc [11]. Benefits of the inclusion of DNA-IL-12 in DNA/viral vector schemes by parenteral routes have been largely explored, specially demonstrating its potency to increment cellular immune responses [12], [13] and also its enhancement control against a pathogenic SIV challenge in the macaque model [14]. Interestingly, with respect to intranasal IL-12 application different reports demonstrated its effectiveness to modulate antigen-specific immune response and to enhance immunization strategies for mucosal vaccines [15], [16]. In a previous reported study it was shown that in an intranasal vaccination using IL-12 plus CTB as adjuvants, both molecules can act in synergy to enhance mucosal and systemic antibodies against HIV-1 glycoproteins [17].

Considering the importance of exploring mucosal immunization regimes in order to find optimal vector combinations and also appropriate mucosal adjuvants in the HIV vaccine development, we conducted the present study in which we analyzed the interaction of DNA-IL-12 and CTB after their mucosal administration in DNA prime/MVA boost intranasal regimes. The novelty of our study reside in that we found a cooperation for IL-12+CTB to improve specific systemic and mucosal cellular immune responses against HIV antigens after DNA/MVA mucosal delivery. We have carried out an exhausted characterization of the specific immune response generated against HIV-1 gp160 antigen as a model. Induced T-cell response was characterized in terms of magnitude, pattern of cytokines secreted, amplitude (cross-reactivity against different HIV Env peptide subtypes), and most importantly in terms of the *in vivo* cytotoxicity capacity. Remarkably, these evaluations were performed at both systemic and mucosal genital tract compartments.

## Materials and Methods

### Cell lines

BHK-21 cells (fibroblast adherent cell-line derived from hamster kidney, ATCC Cat No CCL-10) were maintained at 37°C in a 5% CO_2_ atmosphere in Dulbecco's Modified Eagle's Medium (DMEM, Gibco BRL, USA) supplemented with 2 mM L-glutamine (Gibco BRL), 100 U/ml penicillin (Gibco BRL) and 0.1 mg/ml streptomycin (Gibco BRL) and 10% fetal bovine serum (FBS, Natocor) (DMEM 10%).

### DNA vectors

DNA plasmid (pCR3) expressing gp-120 modified for optimized codon usage (syngp120 mn V3 LAI) from HIV-1 strain IIIB (DNA-EnvB) as previously described [18] was a generous gift of Jürgen Haas (Munich, Germany). The DNA plasmid carrying the sequence coding for IL-12 (expressing murine IL-12, p35 and p40 genes DNA-IL-12) [12], [19] was kindly provided by Dr. Mariano Esteban (Madrid, Spain). To corroborate the expression of the cytokine from the DNA-IL-12 plasmid an assay to quantify the amount of cytokine released into the supernatant of transfected cells was conducted. For this, 293-T cells (human epithelial cells) and 3T3 cells from mouse origin (mouse fibroblasts) were transfected with 5 µg of DNA-IL-12, using Lipofectamine (Invitrogen, USA), following the manufacturer's instructions. The amount of cytokine was quantified in the supernatant by ELISA, 48 h post-transfection (optEIA, BD). Transfected cells produced 228×10^4^ pg/ml (293-Tcells) and 178×10^3^ pg/ml (3T3 cells) of IL-12 in average (versus 700 (293-Tcells) and 1400 (3T3 cells) pg/ml background levels of DNA-empty transfected cells) thus verifying the ability of the vector to express this molecule (data not shown). Plasmids were purified with Endo free Maxi-Prep purification kits (NucleoBond Xtra Maxi Plus EF, Macherey-Nalgen, Germany) using pyrogen-free material and eluted in TE buffer.

### Viruses

MVA recombinant virus encoding the complete IIIB HIV-1 gp-160 (MVA-EnvB) used in this study was previously described [20], [21] and kindly provided by Dr. Mariano Esteban (Madrid, Spain). Amplified viral stocks were grown in BHK-21 cells. Viruses were released from the infected cells by sonication, then purified by ultracentrifugation through 45% sucrose-cushion as elsewhere indicated [22]. MVA-EnvB was titrated by immunostaining of fixed BHK-21 infected cell-cultures with polyclonal serum reactive against VV proteins.

### Mice, immunization protocols, sample collection and processing

Specific pathogen-free (SPF) female BALB/c mice (H-2d) six to eight weeks-old were purchased from the Laboratories of the School of Veterinary Sciences, University of La Plata, Buenos Aires, and then housed in our animal facilities. All experiments were carried out in strict accordance with the recommendations in the Guide for the Care and Use of Laboratory Animals of the National Institutes of Health. The protocol was approved by the Committee of Care and Use of laboratory animals from the School of Medicine, University of Buenos Aires (Permit Number: 508/2009).

The DNA-EnvB (50 µg) prime/MVA-EnvB (10^7^ PFU) boost intranasal immunization scheme was applied in anesthetized mice spaced by 14 days. Both vaccine vectors were diluted in sterile PBS, in 20 µl of final volume. Doses and combination of adjuvants applied in the different immunization schemes are depicted in Table 1. DNA-IL-12 was administered during the priming doses at the indicated dose (50 or 100 µg). Cholera toxin B subunit (CTB, Sigma-Aldrich) and complete cholera toxin (CT, Sigma-Aldrich) were resuspended in sterile distilled water and then diluted in PBS in order to give 10 µg doses. Ten, 30 or 53 days after the last immunization, mice were sacrificed to collect samples. The number of animals of each experimental group was of 4 or 6 animals depending of the experiment.

**Table 1:** Intranasal immunization schemes administered.

Groups a	Prime: Day 0	Boost: Day 14
**None adjuvant**	DNA-EnvB	MVA-EnvB
**CT**	DNA-EnvB + CT (10 µg)	MVA-EnvB + CT (10 µg)
**IL-12_50_**	DNA-EnvB + DNA IL-12 (50 µg)	MVA-EnvB
**IL-12_100_**	DNA-EnvB + DNA IL-12 (100 µg)	MVA-EnvB
**IL-12_50_+CTB**	DNA-EnvB + DNA IL-12 (50 µg) + CTB (10 µg)	MVA-EnvB + CTB (10 µg)
**IL-12_100_+CTB**	DNA-EnvB + DNA IL-12(100 µg)+ CTB (10 µg)	MVA-EnvB + CTB (10 µg)
**CTB**	DNA-EnvB + CTB (10 µg)	MVA-EnvB + CTB (10 µg)

a All groups were immunized with a DNA/MVA immunization scheme by the intranasal route. At day zero mice were primed with DNA-EnvB (50 µg) and 14 days later all groups were boosted with MVA-EnvB (10^7^ PFU/dose). Time as well as quality and quantity of the adjuvants co-inoculated are indicated.

Blood samples were obtained by cardiac punction from anesthetized mice. Blood was allowed to clot 4 hours at room temperature. After leaving samples at 4°C overnight, they were spun down in a microcentrifuge, and sera were removed and stored at −80°C. Vaginal washings were obtained by flushing ten times with 50 µl of PBS into the vaginal canals and it was repeated two times; then samples were spun down to remove cellular debris and frozen at −80°C. Splenocytes and lymphocytes from the genito-rectal associated lymphoid tissue (iliac lymph nodes, ILNs), and nasal-associated lymphoid tissue (cervical lymph nodes; CLNs) were isolated by routine methods. Lymphocytes from urogenital tract were obtained as previously described [5], [23]. Briefly, the urogenital tracts (vagina, cervix, and uterine horns) were aseptically removed, and tissue was longitudinally spliced, cut in very small pieces, and washed with RPMI (RPMI-1640 medium, Gibco BRL) plus 5% FBS. Tissues segments were dissociated enzymatically with a mixture of collagenase type VIII (Sigma-Aldrich) and dispase I (Roche Diagnostics, Germany) in RPMI 5% FBS, with agitation for 30 min at 37°C. After three digestion cycles, lymphocytes were enriched by placing the cell suspension on a discontinuous gradient containing 75% and 40% Percoll (Amersham Pharmacia Biotech, Bucks, U.K.). After centrifugation (1700 rpm, 20 min) the interface layer was harvested, washed, and finally resuspended in RPMIc (RPMI-1640 medium, Gibco BRL,) supplemented with 2 mM L-glutamine (Gibco BRL), 100 U/ml penicillin (Gibco BRL), 0,1 mg/ml streptomycin (Gibco BRL), 10 mM HEPES (Gibco BRL) and β-Mercaptoethanol, with 10% fetal bovine serum (Natocor).

### Peptides

The previously characterized gp160 V3 MHC class I-restricted p18IIIB-I10 RGPGRAFVTI peptide [24], [25] was employed. Potential T-cell epitopes (PTE) peptides of Env protein were obtained from the NIH AIDS Reagent Program. The PTE peptides set represent the most frequent sequences of circulating HIV-1 strains worldwide. The peptides are 15 aa in length. The global PTE peptides cover all PTEs with a frequency equal to or greater than 15% in any one of the subtypes A, B, C and non-ABC. Here, the PTE peptides were pooled in five pools representing the whole protein: Gp120_1_ (aa 1 to 154), Gp120_2_ (aa 155 to 284), Gp120_3_ (aa 285 to 511.), Gp41_1_ (aa 512 to 689.) and Gp41_2_ (aa 690 to 842). In all cases, lyophilized peptides were dissolved in dimethyl sulfoxide (DMSO, Sigma-Aldrich) and stored at −20°C. V3 and peptides pools were used in assays to stimulate T-cells at 2 µg/ml, or at the indicated concentration.

### ELISPOT assays

ELISPOT assays were performed with freshly isolated lymphocytes cultured in RPMIc as described previously [25]. Briefly, 0,125×10^6^ to 10^6^ cells were plated on duplicate or triplicate wells in 96-wells ELISPOT plates (MultiScreen IP plates, Millipore, USA) previously coated with anti-mouse IFN-γ Ab (BD ELISPOT mouse IFN-γ ELISPOT pair) or anti-mouse IL-2 Ab (BD ELISPOT mouse IL-2 ELISPOT pair) The cells were stimulated with individual or pooled peptides. Negative controls were incubated with RPMIc plus 0.04% or 0.08% of DMSO, and cells treated with ConA (1 µg/ml) were included as positive control.

Plates were incubated for 24 h at 37°C in a 5% CO_2_ atmosphere, later washed twice with destilated water and three times with PBS plus 0,05% Tween-20 (PBS-T) and incubated during 16–18 h at 4°C with the corresponding detecting Ab solution (BD ELISPOT mouse IFN-γ/IL-2 ELISPOT pair). Thereafter, plates were washed and incubated during 1 h with Streptavidin-Horseradish Peroxidase (HRP) (BD). Wells were washed and spots were developed by adding 1 mg/ml solution of the substrate 3,3′-diaminobenzidine tetrahydrochloride (Sigma-Aldrich) containing 0.03% hydrogen peroxide. Plates were scanned on an ImmunoSpot reader (Cellular Technology Ltd.) and specific spots were counted using the ImmunoSpot software.

Functional avidity referred as to the activation threshold in response to defined concentrations of exogenous peptide was performed following the protocols previously described [26]. Briefly, limiting peptide dilutions (from 20 µg/ml to 0.00002 µg/ml) were performed and then the sensitizing dose of peptide concentration required to induce a half-maximum IFN-γ secreting cells (SD_50_) in *ex vivo* assays was determined with a sigmoid dose-response curve (GraphPad software). In these Elispot assays the maximal counts for each group were evaluated with comparable E:T ratios for each group. To do this, the number of effector cells/well that was put for each group was selected taking into account the number of spleen cells sufficient to give a response of approximately 200 spots/well.

### Ab measurements by ELISA

ELISA assay was used to determine the presence of Abs against gp160 in serum and vaginal washings following procedures previously described [21]. Purified gp120LAV (Protein Sciences Corp) was employed to coat the 96-well plates at 1 µg/ml. Ab detection was performed after the addition of Biotin-conjugated goat anti-mouse IgG, IgG1, IgG2a or IgA (Invitrogen) followed by the addition of Streptavidin-Horseradish Peroxidase (HRP) (BD). Reaction was developed with the peroxidase substrate TMB (Sigma) and stopped by adding 2 N H_2_SO_4_, absorbance was measured at 450 nm on a Multiskan Plus plate reader (Labsystems, Chicago, Ill).

### T cell-specific cytokine production

Cells were suspended in RPMIc 10% FBS and cultured in triplicate wells (10^6^ cells/well) into 96-well round-bottom plates and stimulated with the V3 peptide (2 µg/ml) or with the recombinant gp120 HIV-1 BaL (NIH AIDS Research and Reference Reagent Program) at 1 µg/ml. Cells stimulated with ConA (1 µg/ml), or medium with the appropriate % of DMSO were the positive and negative controls respectively. After 72 h incubation at 37°C in 5% CO_2_, culture supernatants were stored at −80°C and cytokine analysis was performed using the mouse Th1/Th2 cytokine kit (CBA, Cytometric Bead Array, BD), according to the manufacturer's instructions. The threshold values to consider a positive response was that cytokine-quantities had to be superior to the average values found in negative control wells of each group plus 3SD.

### Simultaneous intracellular cytokine staining (ICS) and cytotoxic activity assessment

Cells (10^6^cells/wells) were stimulated with the V3 peptide at a final concentration of 2 µg/ml during 5 h in 96-well U bottom plates at 37°C in 5% CO_2_ in the presence of the costimulatory antibody anti-CD28 (1 ng/ml; BD Pharmingen), brefeldin A (1 µl/ml BD, GolgiPlug). Monensin (0.7 µl/ml; BD, GolgiStop) and the monoclonal Abs (mAb) anti-CD107a and anti-CD107b both labeled with FITC (CD107a/b-FITC; BD Pharmingen) were added in some experiments, these molecules are degranulation markers that allow the detection of cytotoxic activity of CD8 T-cells [27]. Negative and positive controls consisted of cells stimulated with RPMIc plus 0.08% DMSO, or PMA ionomycin (10 ng/ml phorbol myristate acetate (PMA) plus 250 ng/ml ionomycin (Sigma-Aldrich)) respectively. Afterwards, cells were washed and stained with surface antibodies: CD3-APC and CD8-PerCP (BD Pharmingen), living cells were identified after staining with near-IR-fluorescent reactive dye (LIVE/DEAD, Invitrogen). Cells were incubated for 30 min at 4°C, and then permeabilized and fixed using the fixation/permeabilization kit (BD, Cytofix/Cytoperm). After the permeabilization/fixation step, cells were stained using anti-IFN-γ-PE, TNF-α-PE-Cy7 and IL-2-FITC if applicable (BD, Pharmingen) for 30 min at 4°C in obscurity. After two washes cells were stored at 4°C until being acquired in a BD FACSCanto flow cytometer.

Data acquisition and analysis were done with the BD FACSDiva software. Instrument settings and fluorescence compensation were performed on each testing day using unstained and single stained samples. Stimulated cells stained for surface molecules and isotype matched controls were included in each experiment.

### Proliferation assay

Proliferation assay was performed as previously described [28]. Briefly, splenocytes were resuspended (40×10^6^ cells/ml) in PBS plus 1% FBS and labeled with 1.25 µM of carboxyfluorescein succinimidyl ester (CFSE) (Invitrogen, USA) at 37°C for 8 minutes. The reaction was quenched with FBS and cells were washed with RPMIc. Then, cells were resuspended in RPMIc at a density of 5×10^6^/ml. Cells were cultured in 12 wells plates for 4 days at 37°C and 5% CO_2_ atmosphere with medium only or 2 µg/ml of V3 peptide. Positive controls were stimulated with ConA. After four days, lymphocytes were harvested, counted and washed with RPMIc. Then, cells were stained for 30 minutes at 4°C with anti-mouse CD3-APC, CD8-PerCP, CD44, and CD62L (in memory experiments) (BD Pharmingen) plus viability dye near-IR-fluorescent reactive (LIVE/DEAD, Invitrogen). Cells were then washed and acquired in a BD FACSCanto flow cytometer.

### *In vivo* cytotoxicity

*In vivo* cytotoxicity assays were performed as previously described with brief modifications [29], [30]. Splenocytes from naïve mice were pooled and labeled at 40×10^6^ cells/ml with 1 µM CFSE (CFSE_low_) or 8 µM CFSE (CFSE_high_) (Invitrogen) in PBS plus 1% FBS at 37°C for 8 minutes in the dark and quenched with an equal volume of 100% FBS at room temperature. After washing with RPMIc 10% FCS, CFSE_high_ cells were pulsed with V3 peptide at a final concentration of 5 µg/ml during 30 minutes at 37°C. After 3 washes, cells from each population were mixed together and 20×10^6^ cells were injected i.v. in the different immunized mice groups. Four and 16 h later, mice were sacrificed and individual spleens were analyzed by flow cytometer. In vivo cytolysis was calculated as (1 - (ratio naïve/ratio immune)) ×100, where the ratio  =  percentage CFSE_low_/CFSE_high_.

### Data analysis

Statistical analyses to compare inter-group responses were performed using GraphPad Prism 5 (Graph-Pad Software) employing parametric (Student's t-test; or one-way ANOVA with post-Bonferroni correction), or non-parametric statistics (Mann-Whitney test). For all tests, a p value minor to 0.05 was considered statistically significant.

## Results

### Nasal co-administration of DNA-IL-12 plus CTB cooperate to induce significant enhancement effects in systemic and mucosal cellular immune responses against HIV-1 Env antigen during DNA/MVA immunization schemes

Previous studies have demonstrated the efficacy of the nasal route of inoculation to induce mucosal immune response in vaccination schemes in which MVA vectors were employed [10]. However, neither the analysis of the IL-12 mucosal adjuvant effect applied during mucosal DNA/MVA immunizations nor its effects after its combination with CTB with these vector-based immunizations have been deeply analyzed so far.

Thus, with the aim to evaluate the IL-12 plus CTB action on DNA/MVA regimes administered by intranasal route, different immunization schemes were designed in order to delineate the best DNA-IL-12 dose and to discern the benefits of IL-12 and CTB co-delivery.

Table 1 depicts immunizations applied by intranasal route, in which all mice groups received the same DNA-EnvB dose during priming, but differed whether adjuvants were co-applied or not (control group: without adjuvants). Two different DNA-IL-12 doses were administered with or without CTB, whereas one group received complete CT and the other one CTB only. Fourteen days later, all groups were boosted with 10^7^ PFU/animal of MVA-EnvB, but in those groups which had previously received CT or CTB during priming, the viral boost had the incorporation of CT or CTB respectively. Ten days after the booster dose, the specific cellular immune response generated by the different immunization regimes was analyzed at systemic level (spleen) and also at mucosal lymph nodes, like the CLNs (cervical lymph nodes, draining the nasal mucosa) and the ILNs (iliac lymph nodes, draining the genito-rectal mucosa).

**Figure 1:**
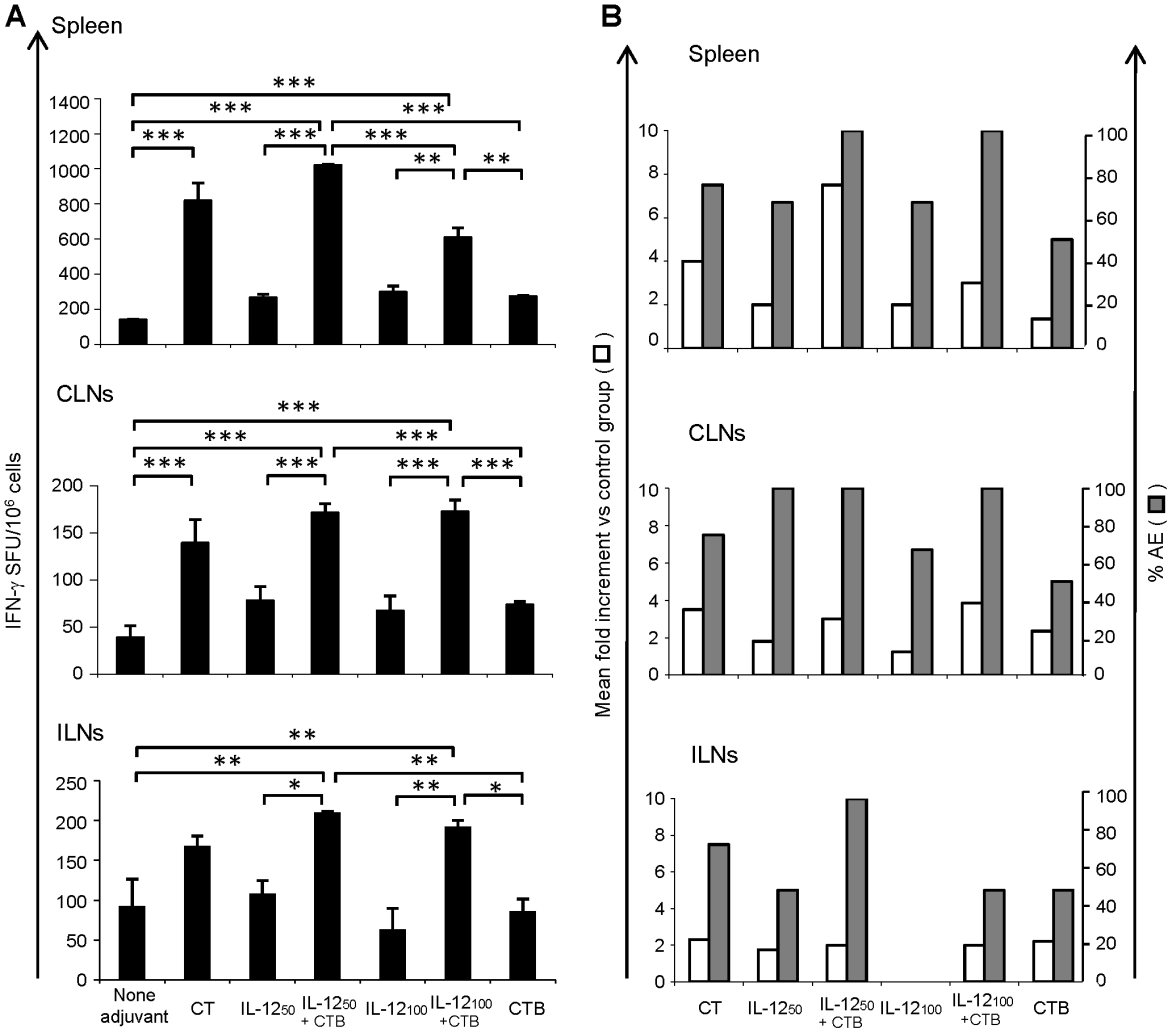
Nasal co-administration of DNA-IL-12 plus CTB cooperate to induce an enhancement effect in the cellular immune responses against HIV-1 Env antigen during DNA/MVA immunization schemes. Groups of four to six BALB/c mice were inoculated as described in the immunization schemes (Table 1). (A) Ten days after the booster dose, specific cellular immune responses (IFN-γ secreting CD8 T-cells) against Env were quantified by ELISPOT using pooled cells from spleen, cervical lymph nodes (CLNs) and iliac lymph nodes (ILNs). Bars represent the average number of specific IFN-γ spots forming units (SFU)/10^6^ cells +SD of a representative experiment from 2 to 6 performed. Each determination was evaluated in duplicate or triplicate and background values from negative control wells were subtracted. Statistical differences between indicated groups: *p<0.05; **p<0.01; ***p<0.001; by one-way ANOVA test and Bonferroni's correction post-test. (B) Summarize of the results obtained in all the experiments performed. Left bars (white) represent the mean fold increments in the magnitude of the specific cellular immune response compared to the control group. Right bars (grey) represent the percentage of experiments with adjuvant efficacy (%AE); %AE: represents a significant increase in the specific cellular immune response induced compared to the control group.

Figure 1 shows data from a representative experiment, in which specific cellular immune responses were evaluated as number of anti-Env (anti-p18IIIB-I10 peptide) IFN-γ secreting cells. In the spleen (Figure 1A upper panel), when DNA-IL-12 was administered alone with the antigen both DNA-IL-12 doses generated similar responses, representing a two-fold enhancement with respect to the magnitude of the response found in the control group (but the enhancements recorded did not reach significant differences). However, when CTB was co-inoculated with DNA-IL-12 at priming and also during the MVA boost dose, significant increases in the numbers of IFN-γ secreting cells could be observed in comparison to the control group and to the respective groups without CTB (IL-12_50_+CTB vs control p<0.001, IL-12_50_ vs IL-12_50_+CTB p<0,001, IL-12_100_+CTB vs control p<0.001 and IL-12_100_ vs IL-12_100_+CTB p = 0,01). Surprisingly, the minor DNA-IL-12 dose plus CTB (IL-12_50_+CTB) generated the highest response representing a mean increment of seven-fold in the magnitude of the response compared to that detected in the control group (Figure 1B) (mean of 1257 vs 185 IFN-γ SFU/10^6^ cells). This result denoted that at least in the spleen, the IL-12 50 µg dose generated a effect co-operative enhancement with CTB that seems to be synergic (mean increment of 7,5-fold). It must be noted that to verify that unspecific plasmid stimulation was not present in the immunization schemes performed along the study, we did an extra immunization experiment in which two mice groups were immunized as the control (non adjuvant) and IL-12_50_ groups and a third group was included (DNA-empty) which received during the priming dose DNA-EnvB (50 µg) +DNAempty (50 µg) (Fig S1A). Ten days after immune response induced at spleen was evaluated by Elispot (Fig S1B) and it can be clearly seen that the magnitude of the response generated in the control group (none adjuvant) that was employed along the different experiments performed in the paper, was comparable to that produced in the mice group i.n. primed with DNA-EnvB(50 µg) +DNAempty (50 µg) (mean of 134 vs 129 IFN-γ SFU/10^6^ cells). On the other hand a significant higher response (p<0.01) was generated in the IL-12_50_ group in comparison to either the non adjuvant or the DNA-empty group.

When responses at mucosal draining lymph nodes were studied, both schemes in which DNA-IL-12 plus CTB were co-inoculated during the priming dose, and CTB was also incorporated during the MVA boost, generated the highest responses in CLNs (p<0,001 vs the control group) and more importantly in ILNs (p<0,01 vs the control group). Then, we considered important to analyze the percentage of experiments in which the application of adjuvants generated a significant enhancement in the cellular immune response (% Adjuvant efficacy (%AE)) and the mean of fold increments found (both parameters in comparison to the control group, (DNA-EnvB/MVA-EnvB i.n immunized without adjuvants) (Figure 1B). Interestingly, these analyses showed that co-delivery of both mucosal adjuvants (DNA-IL-12 and CTB) incremented both measures (%AE and Mean fold increment).

When the specific IFN-γ secreting CD8 T-cells found in the different tissues was evaluated as a whole (Figure 1B), it was observed that the immunization regime applied in the IL-12_50_+CTB group generated the best results in terms of increments afforded and also in relation to the percentage of adjuvant efficacy found (100% of the experiments for all the tissues studied).

### Incorporation of DNA-IL-12 plus CTB in DNA/MVA intranasal immunizations also benefits the humoral arm of the specific immune response

The analysis of the potential antibody enhancement by the combination of IL-12 and CTB after intranasal immunization was previously described for HIV-1 gp120, but in a vaccine formulation in which both the antigen and the cytokine were administered as recombinant proteins [17]. In that report, it was found that IL-12 and CTB act synergistically to enhance both systemic and local mucosal antibody responses to HIV-1 glycoproteins. DNA/MVA immunizations applied in the present study implied a combination of vaccine vectors which predominantly targets and maximizes the T-cellular arm of the specific immune response. Nevertheless, specific Ab targeting was previously reported after mucosal DNA/MVA immunizations. Thus, we decided to investigate the specific humoral immune response found after the different mucosal immunizations applied (Table 1). After 10 days from the boost immunization, IgG anti-gp120 levels were evaluated in sera from individual mice of the different groups. We found that groups which showed significant increments (p<0.05) in the median IgG absorbance values with respect to the control group were the IL-12_50_, IL-12_50_+CTB, CTB, and CT groups (Figure S2A). An in depth examination of the specific IgG subclasses induced, indicated that the presence of CTB during the immunizations incremented the IgG1/IgG2a ratio (Figure S2B). Comparison of the anti-gp120 specific IgG1/IgG2a values between the different groups showed that the higher proportions were found in those groups which received CTB or CT, therefore significant increments (p = 0.03) in the median IgG1/IgG2a values respect to the control group were only found in the groups IL-12_100_+CTB, IL-12_50_+CTB, CTB, and CT.

Afterwards, mucosal specific IgA antibodies were evaluated in the genital tract (vaginal washings samples) quantifying the specific IgA levels. Figure S2C shows the median specific IgA values (referred as IgA fold-increments in relation to pre-immune levels) obtained in the different groups. In general a high dispersion was found, however we observed that for certain groups in which the DNA/MVA immunization was applied with mucosal adjuvants (CT, IL-12_50_, IL-12_50_+CTB and IL-12_100_+CTB), IgA levels were above the control group. In the IL-12_50_+CTB group, specific vaginal IgA levels were significantly incremented reaching 4.12 fold values (p = 0.0146 vs control group) and variability inside this group was minor in relation to the others (median 5.767 [IQ 3.073 to 7.223] vs IL-12_100_+CTB median 11.62 [IQ 1.267 to 22.56]).

Thus these results indicated that improvements of DNA-IL-12 plus CTB nasal co-inoculation during DNA/MVA immunizations were also detected in the specific systemic and mucosal humoral response generated.

### Nasal co-administration of IL-12 plus CTB improved the quality of the mucosal specific cellular immune responses in the genital tract

**Figure 2:**
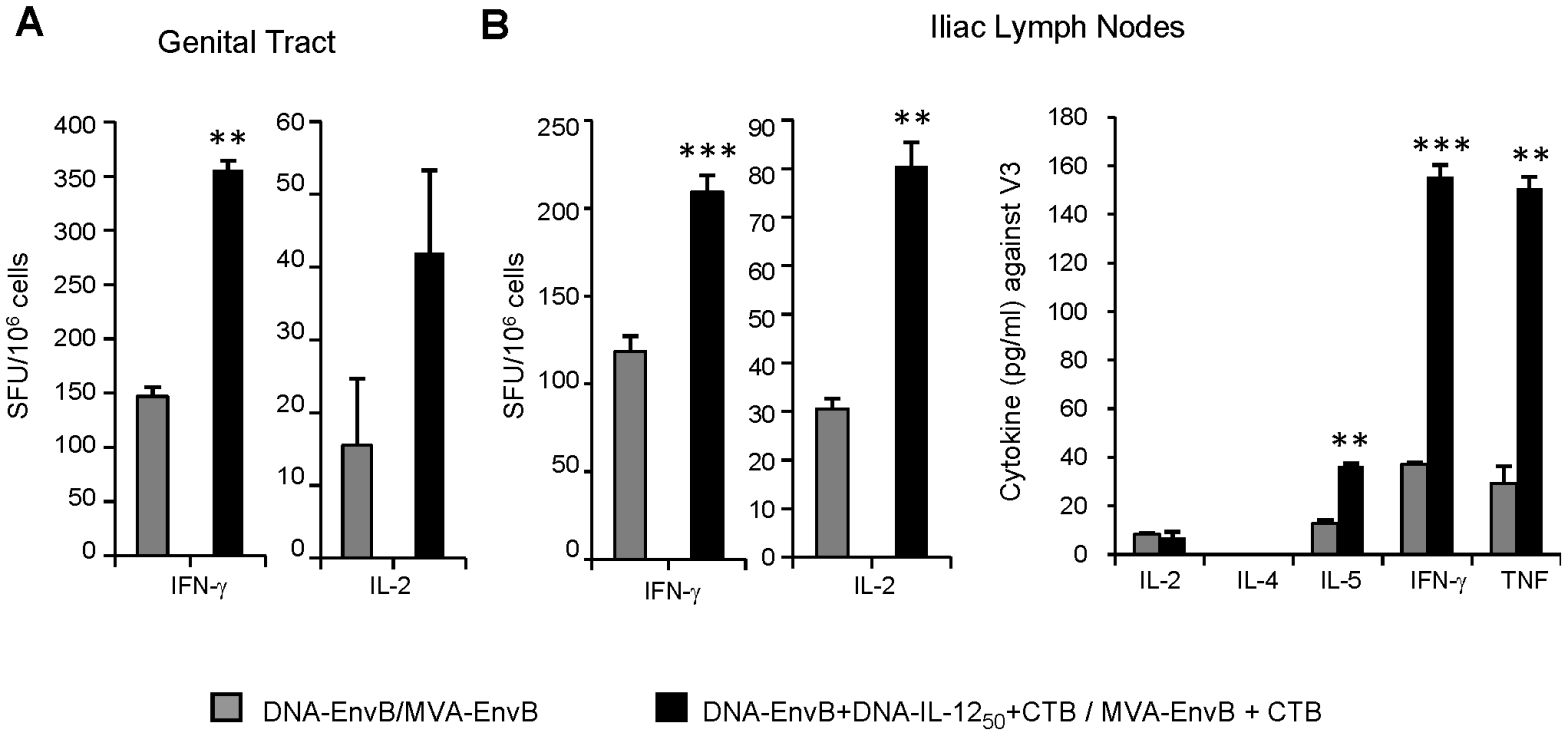
Nasal co-administration of IL-12 plus CTB improved the quality of the mucosal specific cellular immune responses in the genital tract. Groups of six BALB/c mice were immunized following the control: (DNA-EnvB/MVA-EnvB: grey bars) and IL-12_50_+CTB (DNA-EnvB+DNA-IL-12+CTB/MVA-EnvB+CTB: black bars) immunization schemes described in Table 1. Ten days after the MVA booster dose specific cellular immune responses against Env peptide were quantified by ELISPOT assay (IFN-γ and IL-2 secreting CD8+ T-cells) in (A) genital tract and (B) iliac lymph nodes (ILNs, left panel). Data are representative of three independent experiments with comparable results. (B, right panel) Iliac lymph-node (ILNs) cells were stimulated with specific peptide during 72 h and cytokines were measured in culture supernatants using the mouse Th1/Th2 cytokine cytometric bead array (CBA). Bars represent the average values +SD of duplicate determinations from pooled samples. Background values of negative control (RPMIc) were subtracted. Responses were considered as positives if they were above the cut-off values (RPMI +3SD). Statistical differences between groups: **p<0.01; ***p<0.001 by Student's T test.

After the results described above, we decided to evaluate in-depth the specific mucosal immunogenic profile generated after immunization in IL-12_50_+CTB group (details of immunization scheme in Table 1). Thus, cellular mucosal immune responses generated in the genital tract (Figure 2A) and in the genito-rectal draining lymph nodes (ILNs) (Figure 2B) were studied in comparison with the response found in the control group with non adjuvants. As previously shown (Figure 1A) in the ILNs, significant increments in the quantity of specific IFN-γ secreting cells were found, and importantly specific IL-2 secreting cells were also incremented (2.6-fold) (p<0.01) (Figure 2B). More relevant was the finding that specific T-cell responses found at the mucosal effector site (genital tract) were improved in the group which received the adjuvants (IL-12_50_+CTB), as IFN-γ secreting cells were incremented from 147 to 356 SFU/10^6^ cells (p = 0.00164). The number of IL-2 secreting cells detected at this site was lower than IFN-γ but also tended to be higher in the IL12_50_+CTB group. Indeed, it must also be noted that specific IL-2 secreting cells in the spleen of mice from this group were also significantly incremented (from 163 SFU/10^6^ cells in the control group to 331 SFU/10^6^ cells in the IL-12_50_+CTB group, p<0.001, data not shown). To further analyze the specific cytokine pattern secretion by the ILNs lymphocytes, supernatants from lymphocyte-cultures restimulated with the specific Env peptide were analyzed for the production of Th1/Th2 cytokines, by the CBA kit, and as it can be appreciated in the right panel of Figure 2B, not only IFN-γ levels but also TNF (Th1) and IL-5 (Th2) cytokines were significantly elevated in the IL-12_50_+CTB group.

These results clearly showed the benefits of the nasal co-inoculation of DNA-IL-12 and CTB in DNA/MVA mucosal immunization regimes to improve the specific cellular response in the spleen and also in mucosal HIV target sites as the genital tract and iliac nodes which drain the genito-rectal mucosa.

### Improvements on T-cell response generated by DNA-IL-12 plus CTB administration during DNA/MVA immunizations were maintained during the memory phase of the adaptive response

**Figure 3:**
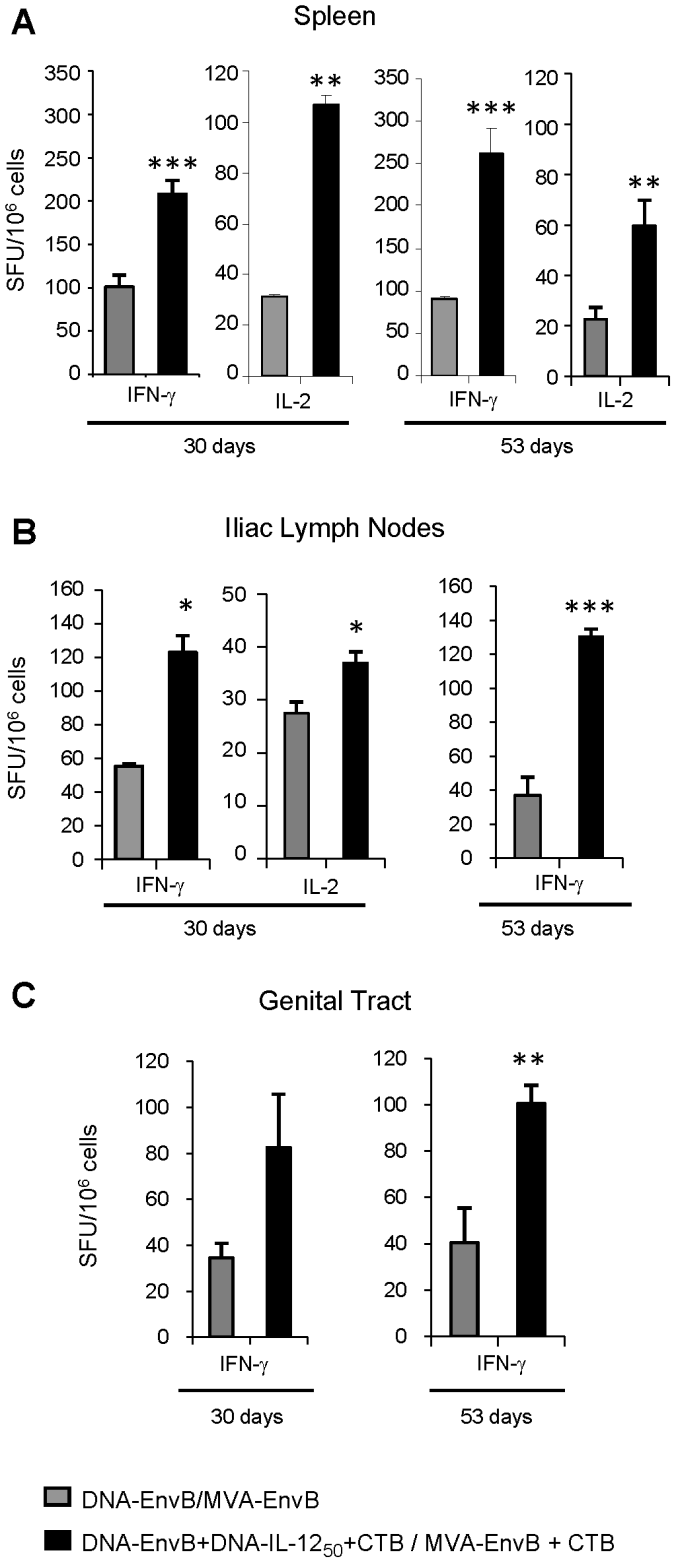
T-cell response improvements afforded by DNA-IL-12 plus CTB administration during DNA/MVA immunizations were maintained during the memory phase of the adaptive response. Thirty or 53 days post- immunization, Env specific IFNγ and IL-2 secreting T-cells were evaluated by ELISPOT using pooled cells from six mice in (A) spleen, (B) iliac lymph nodes (ILNs) and (C) genital tract for DNA-EnvB/MVA-EnvB control group (grey bars), and DNA-EnvB+DNA-IL-12_50_+CTB/MVA-EnvB+CTB group (black bars). Bars represent the average of spot forming units (SFU)/10^6^ cells +SD for duplicate or triplicate wells. Background values of negative control wells were subtracted. Data are representative of three (30 days) and two (53 days) independent experiments. Statistical differences between groups: *p<0.05; **p<0.01; ***p<0.001 by Student's T test.

The results described in the previous section were analyzed after ten days of the booster MVA dose, indicating that the best combination to generate adequate anti-Env B responses at both systemic and mucosal target tissues was DNA-IL-12_50_ dose plus CTB in combination with the antigen (DNA-EnvB) at priming, and CTB plus MVA-EnvB during the booster dose. According to this, our next aim consisted in evaluating whether the improvements afforded in this group were still present at later times post-immunization. Thus, the cellular immune response against Env was analyzed in the different tissues after 30 or 53 days from the booster immunization dose (Figure 3). Cellular responses evaluated in the spleen (Figure 3A) indicated that at later times post-immunization (30 and 53 dpi) mice from the IL-12_50_+CTB group showed two to three-fold increments in the number of specific IFN-γ and IL-2 secreting cells respectively. This result indicated that the increment observed for this group at earlier times (10 dpi, Figure 1A), was maintained despite the contraction of the response. Thus, significant differences were still detected compared to the control group during the memory phase, and also, the pattern found at 30 dpi was maintained at 53 dpi for both CD8 T-cell specific IFN-γ and IL-2 secreting cells. Undoubtedly, an important finding was that the nasal immunization scheme selected resulted efficient to produce significant increments in the memory specific cellular immune responses evaluated in genito-rectal draining lymph nodes (ILNs) (Figure 3B) and in the genital tract (Figure 3C). In the ILNs at 30 dpi, increments detected were similar to those found in the spleen, (2.2 and 1.35-fold for IFN-γ and IL-2 SFU/10^6^ cells respectively), and afterwards at 53 dpi, the magnitude of the response as well as increments detected were nearly maintained (3.5-fold in the IFN-γ response). In the genital tract at these times post immunization we could only evaluate the IFN-γ response, as quantities of specific IL-2 cells were very low, but remarkably in this site, at both 30 and 53 dpi, the IL-12_50_+CTB group showed nearly 2,5-fold increments in the proportion of specific IFN-γ secreting cells with respect to the control mice (at 53 dpi p<0.01).

The results of this part of the study demonstrated that the intranasal DNA/MVA immunization scheme selected (prime: DNA-EnvB+DNA-IL-12_50_+CTB/boost: MVA-EnvB+CTB) maintained at later times significant increments in the specific IFN-γ and IL-2 T-cell responses against HIV Env antigen, in comparison with the control group in which neither IL-12 nor CTB were applied.

### Mucosal IL-12 plus CTB modulation of the T-cell functional profile: Influence on the T-cell capacities of degranulation and specific cytokine pattern production

The importance of T-cell polyfunctionality, defined as those cells that have multiple effector functions, including the degranulation capacity (measured as CD107a/b positive cells) and the production of cytokines/chemokines has been hardly described. In the context of HIV infection and HIV vaccination, several studies have found the association of a specific polyfunctional response as a marker indicative of T-cell efficacy [31]. Specifically, certain polyfunctionality capacities as a higher proportion of HIV-specific cells able to both degranulate and secrete IFN-γ were associated with an improved viral suppression of viral replication during HIV acute infection [32].

**Figure 4:**
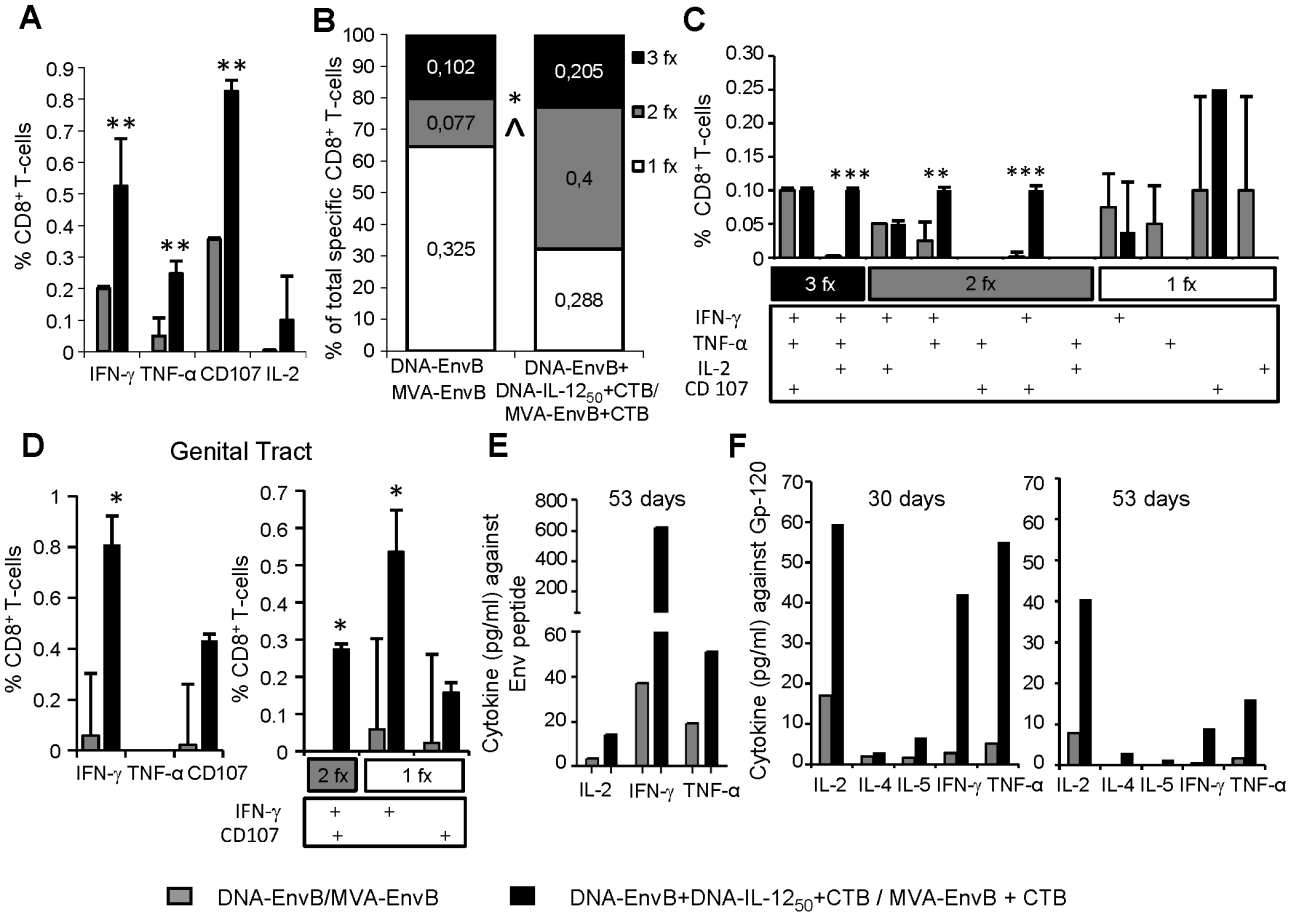
Mucosal IL-12 plus CTB modulates the T-cell capacities of cytotoxicity and specific cytokine pattern production in spleen and genital tract. Quality of the response was evaluated in pooled samples from six mice immunized as DNA-EnvB/MVA-EnvB (grey bars) and DNA-EnvB+DNA-IL-12_50_+CTB/MVA-EnvB+CTB (black bars) groups. (A) Ten days after the MVA boost, the proportion of cytokine secreting (IFN-γ, TNF-α and IL-2) and cytotoxic (CD107a/b) specific CD8+ T-cells against V3 loop were determinated by ICS in splenocytes. (B) Considering the total of specific CD 8^+^ T-cells, percentages of monofunctional (1fx, white), bifunctional (2fx, grey) and trifunctional (3fx, black) cells are represented. (C) Percentages of specific CD8 T-cells detailing the function performed. (D) The same analysis performed in (A) and (C) was done for genital tract cells. The percentage of specific CD8 T-cells able to secrete IFN-γ, TNF-α, or with cytotoxicity capacity (CD107a/b+ cells) and polyfunctionality are depicted in the left and right panel respectively. Bars represent the average +SD for duplicate of pooled cells from six mice. Statistical differences between groups: *p<0.05; **p<0.01; ***p<0.001 by Student's T test (E) 53 days after the boost dose, splenocytes from immunized mice were stimulated with V3 loop peptide during 72 h and cytokines were measured in culture supernatants using the mouse Th1/Th2 cytokine cytometric bead array (CBA). (F) 30 and 53 days after the MVA boost, splenocytes were stimulated with recombinant Gp-120 protein and cytokines were measured as in (E). Bars represent the average values +SD of duplicate determinations from pooled samples. Background values of negative control (RPMIc) were subtracted. Responses were considered as positive if they were above the cut-off values (RPMI +3SD).

Taking these important facts into account, our next aim was to analyze the quality of the response generated by DNA-EnvB+DNA-IL-12_50_+CTB/MVA-EnvB+CTB in comparison with the control group. Thus, the ability of the specific CD8 T-cells, induced after this mucosal immunization, to degranulate and produce different cytokines (IFN-γ, TNF-α and IL-2) was evaluated by an ICS assay. Figure 4A-C shows the analysis of T-cell responses in the spleen ten days after immunization. In concordance with the results described in Figure 1, we found a significantly higher percentage of specific CD8 T-cells able to produce IFN-γ (p<0.01) or TNF-α (p<0.01), or to degranulate (CD107a/b^+^ cells) (p<0.01), in the IL-12_50_+CTB group in comparison to the control one (Figure 4A). Then, the proportion of specific CD8 T-cells exerting one, two or three functions was analyzed (Figure 4B). In the IL-12_50_+CTB group, a significantly higher proportion of specific bi-functional cells was found (in relation to the total specific cells: 45% in IL-12_50_+CTB vs 15% in the control group), implying 0.4% vs 0.077% of total CD8 T-cells (p = 0.022). Then, when polyfunctionality was analyzed taking into account the specific combination of functions, we corroborated that improvements found in the IL-12_50_+CTB group were attributed to a higher proportion of tri-functional CD8 T-cells able to secrete IFN-γ^+^, IL-2^+^ and TNF-α^+^ (p<0.001), and to higher proportions of bi-functional CD8 T-cells that simultaneously produce IFN-γ and TNF-α (p<0.01) and also to bi-functional CD8 T-cells able to degranulate and secrete IFN-γ (CD107a/b^+^ IFN- γ^+^) (p<0,001) (Figure 4C). The same analysis was done with genital tract cells to characterize the functional profile of the specific CD8 T-cells present at the mucosal genital tract site (Figure 4D). The results obtained showed that in the IL-12_50_+CTB group, a significantly higher proportion of cells that produce IFN-γ compared to the control group (DNA-EnvB/MVA-EnvB without adjuvants) was induced (p = 0.038). We also found that specific CD8 T-cells able to degranulate tended to be higher in the IL-12_50_+CTB group, but in none of the two groups we detected TNF-α production. The polyfunctional evaluation of these cells is depicted on the right panel. Importantly, bi-functional cells were only detected in the IL-12_50_+CTB group (IFN-γ^+^ and CD107a/b^+^ cells).

At later times post-immunization (30 and 53 dpi), the Th1/Th2 cytokine pattern secreted in the splenocyte-supernatants was analyzed after the *in vitro* restimulation with either the specific Env peptide (Figure 4E) or the rgp120 protein (Figure 4F). After Env peptide restimulation the analysis employing the CBA kit, revealed that higher levels of IL-2, IFN-γ and TNF were produced in the IL-12_50_+CTB group in consonance with the results found after ICS at earlier times post-immunization (Figure 4A). After gp120 restimulation, as expected for a DNA/MVA based immunization schedule, a marked Th1 pattern was found, which was significantly incremented (IFN-γ, IL-2, TNF) by the co-administration of DNA-IL-12 + CTB during the prime and the re-inforcing of the MVA boost by the co-delivery of CTB. These last results indicated that IL-12 plus CTB not only improved the magnitude of the responses but also benefited the quality of the cellular responses induced evaluated as T-cell polyfunctionality and variety of cytokines secreted.

### Impact on T-cell quality measured by its proliferative potential and its ability to recognized cross-reactive peptides

**Figure 5:**
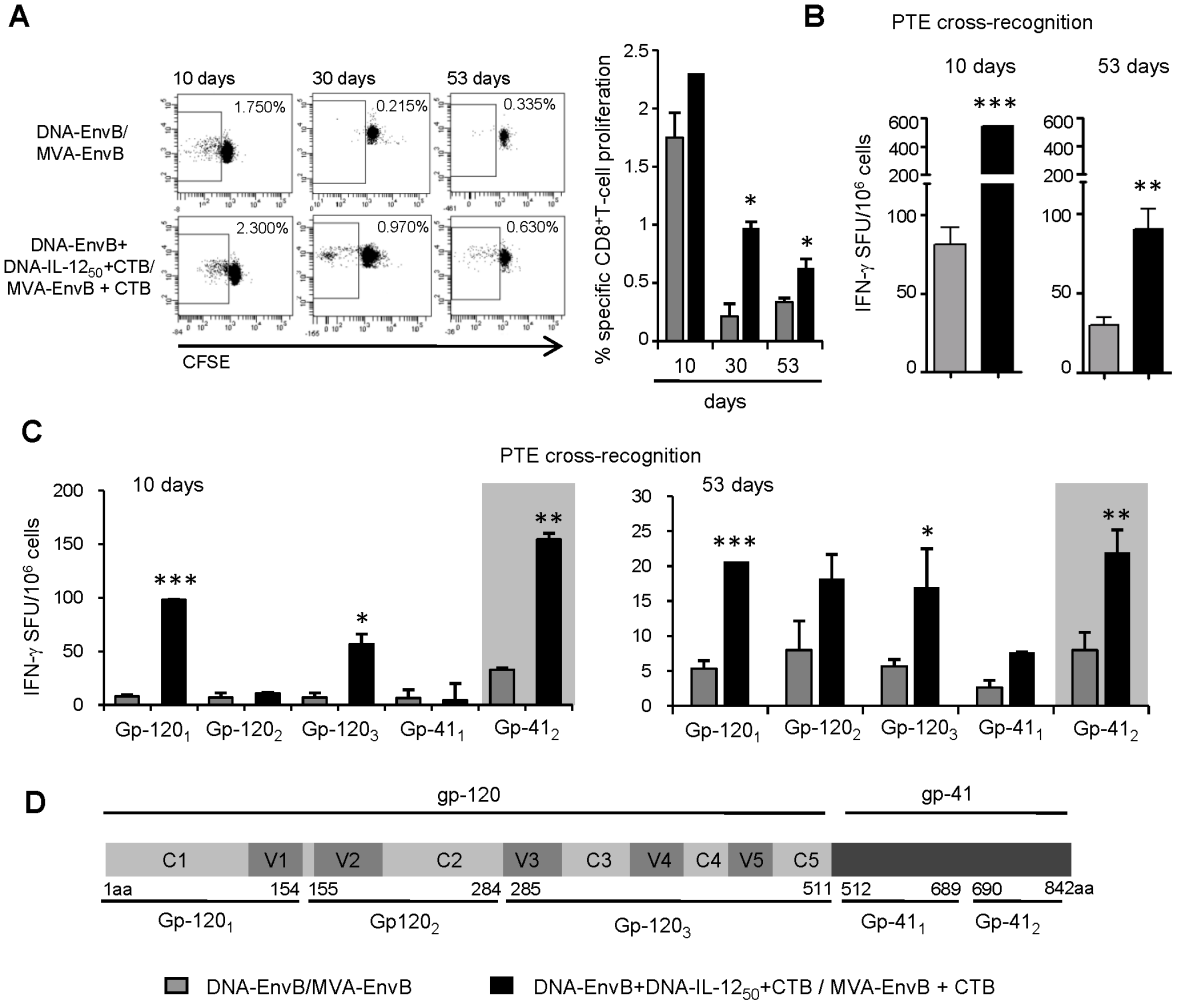
CD8^+^ T-cell quality measured by its proliferative potential and its ability to recognized cross-reactive peptides. Specific cellular immune responses were analyzed at 10, 30 or 53 days after the immunization in groups of six BALB/c mice. Pooled splenocytes of DNA-EnvB/MVA-EnvB (grey bars) and DNA-EnvB+DNA-IL-12_50_+CTB/MVA-EnvB+CTB (black bars) groups were evaluated. (A) CFSE-labeled cells were stimulated *in vitro* during 4 days with the specific V3 loop peptide and then stained with surface antibodies (CD3 and CD8). Proliferating CFSE low CD8 T-cells was determined by flow cytometry. A representative dot plot is depicted (left panel) and percentage of proliferating cells were analyzed (right panel). (B) Ten and 53 days after immunization, splenocytes were stimulated with different pools of Env PTE peptides and evaluated by ELISPOT. Bars represented the accumulated number of IFN-γ spot forming units (SFU)/10^6^ cells for the total PTE peptide-pools. (C) Magnitude of the response against the individual PTE peptide-pools is depicted. (B) and (C): Data are representative of two independent experiments. Bars represent the average +SD for duplicate samples. Background values were subtracted in each case. Statistical differences between groups: *p<0.05; **p<0.01; ***p<0.001 by Student's T test. (D) Scheme indicating the gp160 region included in the different PTE peptide pools.

We also analyzed the specific proliferative potential of the T-cell response generated, for this we performed CFSE-based proliferation assays in which experiments were carried out at ten dpi and also during the memory phase of the adaptive response (Figure 5A). We found that the percentage of Env-specific proliferative CD8 T-cells was incremented in the IL-12_50_+CTB group. At 10 dpi a tendency to higher percentage of proliferative cells was detected in the group with adjuvants vs the control group (2.3% vs 1.75%) but more importantly, the difference between both groups was much pronounced at later times (30 dpi and 53 dpi), when cells from IL-12_50_+CTB mice showed an increase in the specific proliferation capacity in up to four-fold in relation to that observed in the control group (30 dpi, 0.97% vs 0.21%, p = 0.012).

An immune response directed against HIV with higher breadth, having the ability to recognize multiple epitopes and/or different variants of the targeted peptides, is a desired feature to be hold by a vaccine formulation. Considering this fact as an important skill that an immunization schedule must have, we proceeded with the analysis of the cross-reactivity against a pool of peptides from gp160 of different HIV subtypes. For this, we performed an ELISPOT assay using the Env PTE (Potential T-cell Epitopes) peptide set, in which a total of 480 peptides representing A, B, C and non-ABC HIV-1 gp160 subtypes were included. In this case, the peptide set was divided into five pools: Gp120_1_ (aa 1 to 154) including the first constant (C) and variable (V) structural domains of the protein (C1 and V1), Gp120_2_ (aa 155 to 284) including V2 and C2, Gp120_3_ (aa 285 to 511) spanning from V3 to C5 inclusive, and finally the peptides that comprise the Gp41 portion of the protein were divided into two pools: Gp41_1_ (aa 512 to 689) and Gp41_2_ (aa 690 to 842) (Figure 5D). Results from PTE ELISPOT assays performed 10 days after the last immunization dose, showed that a significantly higher total response (of up to six-fold, p<0.001) against the gp160 PTE peptides (evaluated as the sum of SFU/10^6^ cells detected against each of the five Env peptide-pools) was detected in mice which received the adjuvants (IL-12_50_+CTB) (Figure 5B left panel). Then, when the response was studied in a more in-depth analysis taking into account the responses against the different peptide-pools in which the Gp160 PTE peptides were divided, we verified that the main differences between both groups were detected in the response directed against the Gp120_1_, Gp120_3_ and Gp41_2_ pools (Figure 5C left panel).

Data from two independent experiments showed significant differences against Gp41_2_ pool in both experiments (p<0.01) whereas responses against Gp120_1_ and Gp120_3_ pools resulted significantly higher in the IL-12_50_+CTB group (p<0.001 and p<0.05 respectively) in 1 out of 2 experiments. More importantly was the fact that at 53 dpi, during the memory phase of the response, the pattern of the responses observed at earlier times (Figure 5B) was conserved. Thus, the total response against the PTE peptide-pools comprising the whole Gp160 sequence was significantly higher (three to four-fold higher) in the IL-12_50_+CTB group vs the control mice (p<0.01) (Figure 5B right panel). Then, when we considered the T-cell responses against the different PTE peptides pools, significant differences were found against the same regions detected previously at 10 dpi (Gp120_1_, Gp120_3_ and Gp41_2_) (Figure 5C right panel).

These results demonstrated that the co-administration of IL-12_50_ plus CTB during the priming and CTB during the booster, also improved the specific proliferative potential of the CD8 T-cell response induced. Furthermore, another important advantage was the fact that this immunization scheme induced T-cells that were able to recognized heterologous peptides to the antigen expressed from the vaccine, thus enhancing the breadth of the cellular response induced.

### Improvements in T-cell functional avidity and *in vivo* T-cell specific killing

**Figure 6:**
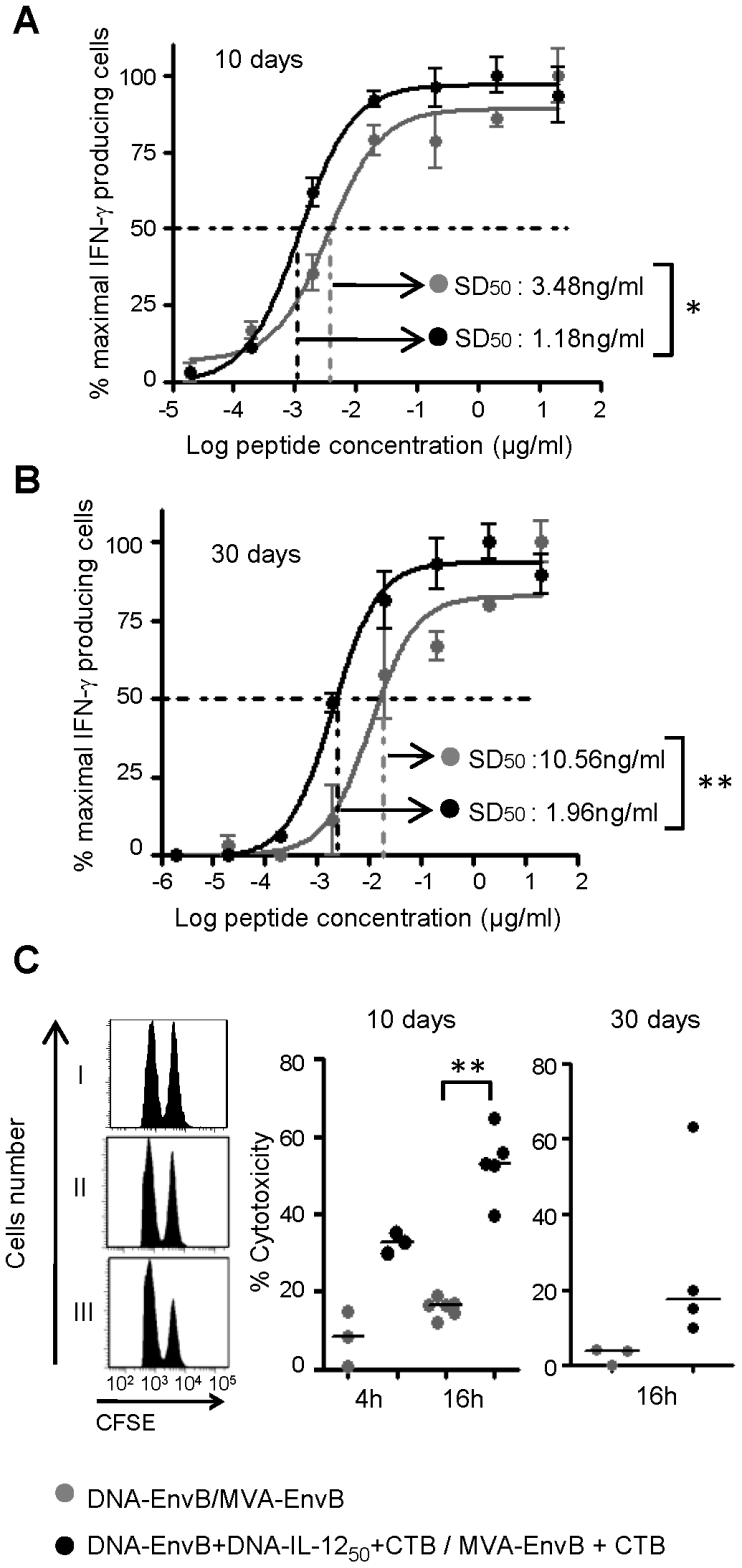
Study of T-cell functional avidity and in vivo T-cell specific killing activity. Pooled splenocytes from control and IL-12_50_+CTB groups (three to six mice/group), were analyzed for functional avidity of the V3- loop specific T-cells at (A) 10 and (B) 30 days after immunization, by ELISPOT using serial dilutions of the peptide from 20 to 0.00002 µg/ml for triplicate wells. Data represents the average percentage of the maximal response. SD50: sensitizing dose of peptide required to yield 50% of maximal T-cell triggering IFN-γ production. (C) *In vivo* cytotoxicity was analyzed adoptive transfer of control CFSElow and Env-peptide pulsed CFSEhigh dye cells. At 10 and 30 days after immunization, individual spleens of mice from naïve (I), DNA-EnvB/MVA-EnvB (II) and DNA-EnvB+DNA-IL-12_50_+CTB/MVA-EnvB+CTB (III) groups were analyzed at the indicated times after adoptive transfer. A representative histogram is depicted in the left panel. Dots represent each analyzed mice. Lines represent median Statistical differences between groups: *p<0.05; **p<0.01 by Mann-Whitney test.

Another feature of the T-cell response that correlated with effectiveness in their effector functions is the cellular avidity for the antigen. Regarding HIV infection, recent studies demonstrated that individuals with the capacity to control the infection harbor CD4 T-cells with the intrinsic ability to recognize minimal amounts of Gag antigen [33], and also that the presence of high-avidity Gag-specific and HLA-B restricted CTL response correlated with viral suppression *in vivo* [34]. Taking into account this background information, we analyzed the T-cell avidity induced against the Env V3 peptide after applying the immunization scheme of the IL-12_50_+CTB group. For this, we performed ELISPOT assays at different peptide concentrations, making serial dilutions. In these experiments, the T-cell avidity power is reflected by the capacity of T-cells to recognize its specific peptide-ligand at low concentrations, and it is expressed as the peptide concentration that is required to detect 50% of the maximal specific T-cell response: SD_50_ (sensitizing dose of peptides required to yield 50% of maximal T-cell triggering IFN-γ production). Interestingly, T-cells from the group in which IL-12 and CTB were included, showed a significantly higher avidity against the Env V3 peptide, thus the SD_50_ resulted three times lower for the IL-12_50_+CTB group compared to the control group (1.18 vs 3.48 ng/ml respectively p = 0.0136) indicating a superior capacity to detect lower quantities of the specific antigen (Figure 6A). More important was the fact that we could demonstrate that the improvements in the T-cell avidity properties observed in the group with adjuvants were still present after 30 days of the immunization schedule, and at this time differences between both groups were enhanced. Figure 6B depicts the curves for the percentages of maximal IFN-γ secreting cells vs the concentration of the specific peptide-ligand, found at 30 dpi, and it can be seen that whereas in the IL-12_50_+CTB group the SD_50_ value was nearly maintained in relation to that observed at 10 dpi (Figure 6A) (1.95 ng/ml (30 dpi) vs 1.18 ng/ml (10 dpi)) (p>0.05), the SD_50_ value for the control group was significantly incremented (10.56 ng/ml (30 dpi) vs 3.48 ng/ml (10 dpi)) (p = 0.026). Therefore, during the memory phase of the response the difference between the T-cell affinity capacities for both mice groups was increased, being the SD_50_ value for the IL-12_50_+CTB group 5.4 times minor than that observed in the control one (p = 0.0035).

Finally, we considered relevant to perform an assay whereby we could evaluate *in vivo* the quality of the T-cell response induced. For this purpose, we selected an assay to measure the level of specific killing capacity of the CD8 T-cells evaluated *in vivo*, considering a solid way to evaluate the quality of the response, and to corroborate if differences evaluated by *in vitro* functional assays (as IFN-γ secretion by ELISPOT assay, ICS or avidity curves) are readily reproduced by an *in vivo* functional assay. The steps applied in the development of this assay are described in detail in the methods section. Briefly, ten days after immunization, mice from the different groups (control, IL-12_50_+CTB and naïve) were intravenously injected with CFSE labeled control and Env peptide-pulsed target spleen cells. After 4 h and 16 h animals were sacrificed and specific lysis was analyzed by flow cytometry. Figure 6C left upper panel shows representative data of CFSE histograms observed in the naïve mice, in which the two peaks of the histograms corresponding to CFSE_high_ (peptide-pulsed) and CFSE_low_ (un-pulsed) target-cell populations have similar sizes, indicating that in naïve mice after 4 h or 16 h post inoculation similar quantities of both target-cell populations were recovered (Figure 6C, left panel). On the contrary, in mice from immunized groups (middle and inferior panels) the histograms corresponding to CFSE_high_ (peptide-pulsed) cells were diminished in relation to the CFSE low histograms, being this difference more pronounced for mice of the DNAEnvB+DNAIL-12_50_+CTB/MVA-EnvB+CTB immunized group (IL-12_50_ +CTB). Percentage of specific *in vivo* cytolisis was analyzed at 10 dpi and 30 dpi. After 10 days cytotoxicity was evaluated at 4 h and also 16 h post-transfusion (pt) of the CFSE dyed cells. As it can be appreciated in the middle panel of Figure 6C, in the IL-12_50_+CTB group the percentage of cytoxicity levels detected 4 h and 16 h pt of cells was nearly 3.9 and 3.2 times higher than the percentage observed in the control group. At 4 h pt although higher values of percentage of cytotoxicity were detected in the group with adjuvants, differences between both groups did not reach statistical significance. Later at the time point of 16 h pt, median values of cytotoxic activity found in IL-12_50_+CTB mice group were significantly higher compared to those detected in the control mice (median values of 53.03% vs 16.37% p = 0.0043). When the *in vivo* killing assay was performed during the memory phase of the adaptive T-cell response (Figure 6C right panel) we found that *in vivo* cytotoxic activity was still detected, and the comparison between both mice groups showed median cytotoxicity values of 3.51% (control) vs 17.51% (IL-12_50_+CTB) (p = 0.057).

These last experiments demonstrated a direct correlation between CD8 T-cell effector functions exerted *in vivo* and specific T-cells assays previously analyzed *in vitro*.

### Memory T-cell subpopulations induced after DNA/MVA immunization schemes showed a distinctive phenotype in which IL-12+CTB co-administration generated an enhancement of T-cells from earlier stages of differentiation

**Figure 7:**
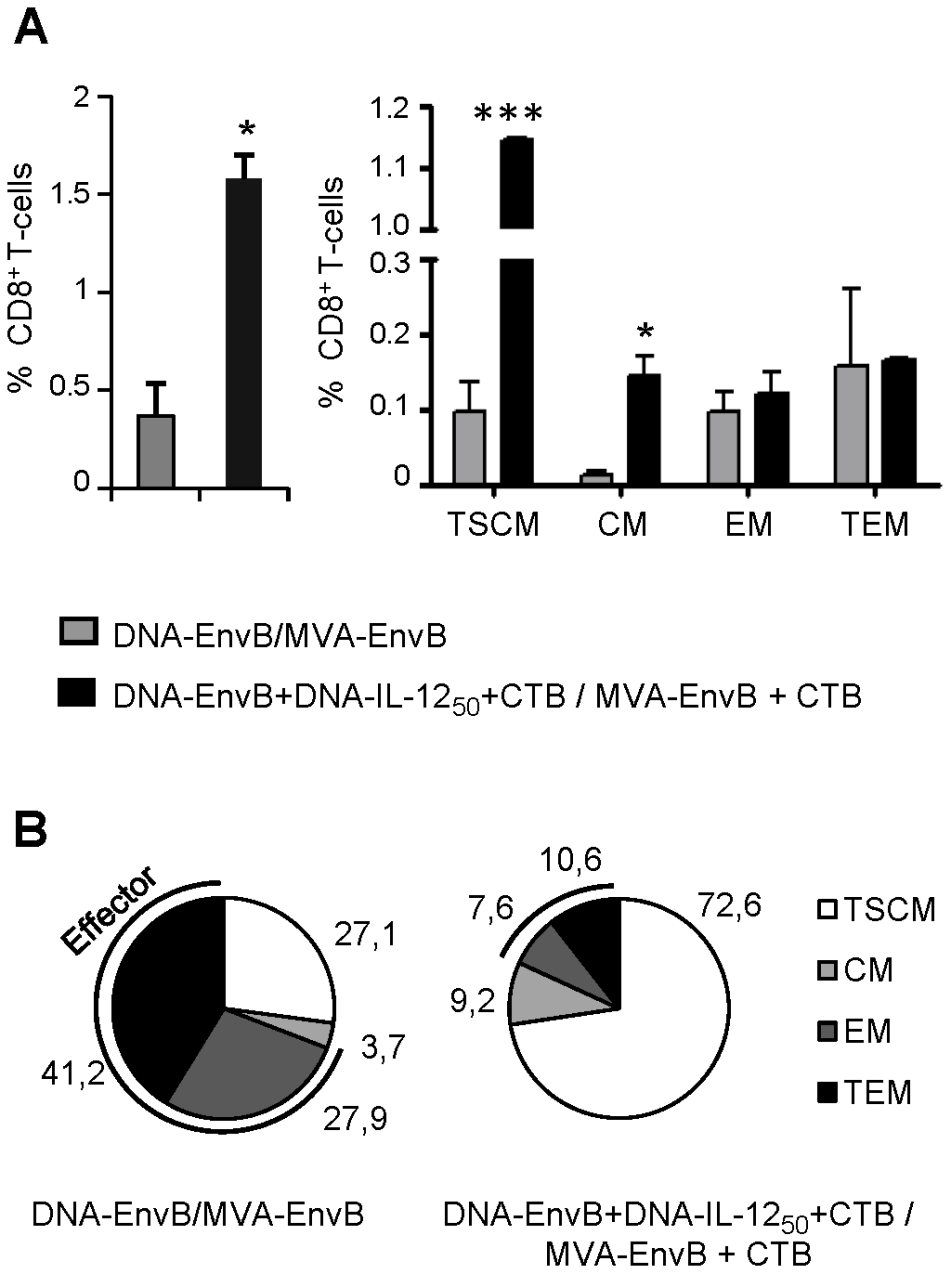
Characterization of specific memory T cell subpopulations induced. Thirty days after immunization, pooled-splenocytes of six BALB/c mice were CFSE- labeled and stimulated with V_3_-peptide during 4 days. Then, cells were stained with surface marker (CD8, CD44 and CD62L) and analyzed by flow cytometer. CD8 T-cells with specific proliferative capacity (evaluated by the CFSE dilution assay) were classified in four memory subpopulations. (A) Bars represent the percentage of specific CD8 T-cell with a phenotype compatible with: T stem cells memory (T_SCM_; CD44L-CD62L+), T central memory (T_CM_; CD44+ CD62L+), T effector memory (T_EM_; CD44+ CD62L-) and T terminal effector (T_TEM_; CD44- CD62L-). (B) Pie charts show the percentage of specific CD8 T-cells expressing the indicated phenotype. Statistical differences between groups are indicated: *p<0.05; ***p<0.001 by Student's T test.

Finally, we proceeded to characterize more in depth the phenotypic pattern of the T-cell memory response induced in order to define if the improvements achieved after the co-administration of IL-12 and CTB were accompanied by differences in the specific T-cell memory sub-populations generated. To do this, DNAEnvB+/MVA-EnvB and IL-12_50_+CTB groups were immunized as in previous experiments, and 30 days later T-cell specific responses were analyzed by ICS and by their specific proliferative capacity with CFSE-based assays. By the co-staining of CD8 T-cells with surface markers, T-cells that responded to the antigen (and are therefore memory cells) were grouped into four categories: “early memory or T stem cells memory” (TSCM: CD44- CD62L+), central memory (CM: CD44+ CD62L+), effector memory (EM: CD44+ CD62L-), and terminal effector cells (TE: CD44- CD62L-). In these experiments, splenocytes were restimulated during 4 days with the specific peptide or with RPMI only, in order to evaluate the level of specific proliferation by the dilution of the CFSE staining. Then, the distribution of the CD8 T-cell subpopulations was analyzed by flow-cytometer inside the cells that showed proliferation against the specific stimuli. It can be seen that in the IL-12_50_+CTB group the percentage of CD8 T-cells that proliferated against the specific peptide was significantly incremented (nearly four-fold) with respect to the values observed in the DNA-EnvB/MVA-EnvB control group immunized without adjuvants (Figure 7A). When the distribution of specific memory T-cell populations was compared between both groups, it was notably revealed that mice immunized with the IL-12_50_+CTB scheme showed significantly higher proportions of CD8 specific cells with CM (0.1455% vs 0.013%; p<0.02) and with TSCM phenotypes (1.14% vs 0.098%; p<0.001) (Figure 7A). Figure 7B illustrates the distribution of the antigen-specific cells among the different maturation stages for the two mice groups. It can be clearly seen that after the IL-12+CTB immunization the fraction of cells with an effector phenotype (EM plus TE) was significantly reduced (69% in control vs 18% in IL-12+CTB). Thus, the T-cell phenotypes that resulted significantly incremented after the DNA-IL-12 plus CTB co-administration were associated with T cells from earlier stages of differentiation (TCM and TSCM-like cells).

## Discussion

In this study we analyzed the feasibility and efficacy of the incorporation of DNA-IL-12 and CTB as mucosal adjuvants during the administration of DNA/MVA vectors by intranasal route, employing vectors expressing the gp160 HIV-1 antigen as a model. The results described show that DNA-IL-12 plus CTB can be effectively employed acting as mucosal adjuvants during DNA prime/MVA boost vaccine doses applied by intranasal route. The importance of the results presented here relies in two original relevant findings: a) the capacity of IL-12 and CTB when administered by intranasal route to cooperate in their adjuvant action enhancing both the magnitude and quality of the cellular immune responses induced at systemic tissues (in the spleen) and mucosal sites (CLNs, ILNs and genital tract), b) the description of a new mucosal adjuvant-combination, with potential clinical application, that resulted extremely effective to improve the quality and magnitude of the cellular responses generated by a DNA/MVA mucosal immunization.

The relevance of expanding the knowledge about strategies related to mucosal vaccines is a worldwide concern, as most pathogens access the body through the mucosal membranes and so effective vaccines with the ability to protect at these sites are needed. However, after the oral polio vaccine few new mucosal vaccines have been launched afterwards. One of the reasons for this is the absence of safe and effective mucosal adjuvants [8]. Regarding HIV vaccines, mucosal surfaces are not targeted by most of the current vaccines on clinical trials, except for isolated cases [35].

The delivery of HIV antigens by the DNA prime/MVA boost strategy has been largely probed and now it is being employed in diverse ongoing clinical trials. The application of these antiHIV/SIV vaccine regimes by intranasal route were previously performed in murine and macaque models [5], [7]. Significantly in one of these studies it was demonstrated that nasal DNA/MVA SIV vaccination provides more significant protection from progression to AIDS than a similar intramuscular vaccination [7].

In the designing of mucosal vaccine strategies, the selection of an appropriate adjuvant is crucial in order to generate an effective mucosal immunization. In the present study, we hypothesized that intranasal DNA/MVA immunization may be improved by the inclusion of IL-12 and CTB as adjuvants. The benefits of the co-administration of these molecules by intranasal route have been previously explored, but with a different immunization strategy in which a recombinant protein was administered, and only it was analyzed the capacity to improve the humoral arm of the response [17].

Previous studies have demonstrated that CTB can act as an efficient mucosal adjuvant to enhance both mucosal and systemic antibody responses [36]. Additionally, CTB was shown to be efficient at promoting MHC class I cross-presentation of exogenous antigens by murine dendritic cells, these features were demonstrated with antigens conjugated to or simply mixed with CTB [37]. However, a novel finding of the present study is to demonstrate the intranasal mucosal adjuvant effect of CTB to enhance CD8 T-cell responses at a distal mucosal site as the genital tract. In a previous study in which it was also shown a CTB T-cell enhancing effect at the genital tract after its administration from a distal mucosal route (sublingual delivery), the CTB effect alone cannot be correctly evaluated because even when it was coupled to the Pol peptide antigen, the complete cholera toxin (CT) was also co-administered during immunizations [38]. A similar fact occurred in another work in which a fusion protein consisting of the cholera toxin B subunit and a 36-residue peptide of the conserved membrane proximal ectodomain of gp41 (CTB-MPR649–684) was analyzed using different mucosal routes in relation to its potential to induce either systemic or mucosal anti-MPR649–684 antibodies. In that study, various immunization regimens were examined but mucosal delivery of CTB-MPR649–684 was always accompanied with CT [39].

In relation to IL-12, its administration by mucosal routes was previously described to be effective in the enhancement of cellular immune responses at both systemic and mucosal sites [40], [41]. However, the interaction effects of IL-12 plus CTB administered by intranasal route has not been analyzed in depth. Plasmid encoded IL-12 has proven to be a promising candidate adjuvant, enhancing cellular immune responses when applied during DNA prime-viral vector boost vaccine regimens against HIV or SIV antigens [12], [28], [42]. Interestingly, in a recent preclinical macaque experimental design it was demonstrated that systemic immunization with DNA-IL-12 in a DNAprime and recombinant Ad boost vaccination generated an enhanced control against a pathogenic low-dose intra-rectal SIVmac239 challenge [42].

Here we demonstrate that by the co-delivery of the non-toxic CTB mucosal adjuvant with DNA-IL-12 during the intranasal DNA/MVA vaccine regimen, an enhancement of the magnitude of the specific CD8 T-cell responses is generated not only in systemic tissues (spleen) but also in mucosal lymph nodes proximal to the immunization site (CLNs) and in those draining genito-rectal tissues (ILNs). In the spleen we observed that when IL-12 was administered alone generated a two-fold increment, but when CTB was co-administrated with the DNAprime and MVAboost doses, a co-operative enhancement was detected of up to seven-fold increments (IL-12_50_ dose) in the magnitude of the response compared to that detected in the control group (mean of 1257 vs 185 IFN-γ SFU/10^6^ cells). Undoubtedly, a significant finding was to demonstrate an improvement of the specific cellular response at the genital tract, which functions as a crucial mucosal effector site acting as a barrier against the virus. Moreover, the improvements detected during the acute phase of the immune response were maintained during the memory phase, where we analyzed the specific immune response induced in the spleen, ILN and genital tract up to 53 days from the last immunization.

At early times post-immunization we also evaluated levels of anti-Env specific antibodies generated. Thus, although DNA/MVA immunizations applied in the present study implied a combination of vaccine vectors which predominantly targets and maximizes the T-cellular arm of the specific immune response, results depicted in Figure S2 indicated that improvements of IL-12 plus CTB nasal co-inoculation were also detected in the specific systemic and mucosal humoral response generated.

Evidences indicate that the induction of specific CD8 T-cells at the mucosal portal of entry of the virus is crucial to slow down the initial HIV replication [43], however from the multiple mucosal HIV vaccine studies performed until now only a minority of them analyzed specific cellular responses directly at the mucosal effector site of the genital tract. As an example, in an interesting paper published last year the authors described an original mucosal HIV-1 vaccine strategy based on IL-13Ra2, where the strategy demonstrated to induce CD8 T-cell mucosal enhancements in diverse functions. However, this effect was only analyzed in mucosal draining lymph nodes as the ILNs and PPs, and as mucosal effector site they only evaluate responses at the lung [44].

Analysis of the modulation of the T-cell functional profile showed that one of the improvements detected was that the specific T-cell responses generated showed a higher polyfunctionality, with capacity to secrete a broader spectrum of cytokines (Figure 2–4). Remarkably, genital tract CD8 T-cells were also modulated after the intranasal DNA/MVA IL-12+CTB scheme, in the sense that a higher proportion of cells able to produce IFN-γ and CD107a/b+ and of IFN-γ/CD107a/b bi-functional cells were found (Figure 4D).

We have recently published a study in which we analyzed the impact of the incorporation of GM-CSF and IL-12 during DNA/MVA systemic immunizations against the HIV-1 Nef antigen. The results obtained indicated that the incorporation of DNA-IL-12 in DNA/MVA vaccination schemes produced the best results in terms of improvements of T-cell response key properties such as breadth, cross-reactivity and quality (avidity and pattern of cytokines secreted) [28]. In the present study, similar effects were found when IL-12 was co-inoculated with CTB by intranasal route, thus in addition to its improvements with respect to pattern of cytokines secreted and polyfunctionality, we also found a higher specific proliferative level and perhaps more relevant was the finding of a stronger CD8 T-cell avidity (Figure 5A and Figure 6A). T-cells with high functional avidity respond to very low antigen doses, in relation to the potential impact of mucosal vaccinations on T-cell avidity, previous studies indicated the superiority of a mucosal (intranasal) poxvirus-based immunization in relation to a systemic (i.m) one to generate higher avidity CTL responses [45]. Here, although mucosal vs systemic immunizations were not compared, we could demonstrate the capacity of DNA-IL-12 in conjuction with CTB to augment the functional avidity of the CD8 T-cell response induced after its intranasal delivery in DNA/MVA immunizations. Remarkably, improvements in T-cell avidity during the memory phase indicated that the SD_50_ value for the IL-12_50_+CTB group was 5.4 times lower than that observed in the control (differences between both groups were significantly different (p = 0,0035) (Figure 6B). In relation to the influence that may have the application of cytokines or adjuvants during immunization on the avidity of the specific T-cells clones that are induced, earlier studies have described that IL-12 can promote an enhanced synapse formation between APCs and T-cells, leading to the recognition of weak peptides against which T-cells would be normally unresponsive [46], or that the generation of high avidity effector CTLs is IL-12 dependent [46], [47]. With respect to CTB and its effects on T-cell avidity there are no reports in which this topic has been studied until now.

Another improvement of the IL-12 plus CTB in DNA/MVA nasal immunizations that we could verify along this study was that the response induced showed a higher breadth evidenced by a higher cross-reactivity against Env peptides of different subtypes. Undoubtedly, the induction of a cellular immune response of high breadth able to recognize Env proteins from different HIV subtypes is a desired characteristic for an HIV vaccine. Even more, we had recently described a similar IL-12 improvement for Nef antigens [28].

Finally we performed an *in vivo* CTL assay in order to do a more profound analysis of the CTLs capabilities of the immune response generated [30], the disadvantages that perhaps *in vitro* and *ex vivo* analysis may have is that they are less informative than *in vivo* assays, since they do not take into account the complexity of the microenvironment where the immune response occurs, and therefore do not always reproduce what occurs *in vivo*. Thus, considering these facts our results demonstrating the superior *in vivo* specific cytotoxicity in mice from the IL-12_50_+CTB group, contribute to do a more comprehensive analysis highlighting the relevance of the innovative mucosal DNA/MVA immunization scheme presented in this work. An important point to denote is that the level of specific cytolytic capacity of CD8 T-cells evaluated *in vivo*, can be considered in a certain manner an indirect measure of the potential protective capacity of the CD8 T-cell immunity generated. This concept was previously demonstrated in the previously published work of Zhongxia Li et.al, [48], in which the authors demonstrated a direct correlation between high levels of in vivo cytolytic activity against Gag-peptide-labeled target cells and the protection capacity against a rVACV-gag virus. But as in the present study we have applied a rMVA boost in the immunization scheme, the anti-vaccinia virus immunity generated preclude the use of the useful rVACV challenge model. Another notion that is important to take into account is that from the different T-cell effector functions, the cytolitic activity of CTLs play a preponderant role during SIV/HIV pathogenesis. This concept is supported by biologically-directed models that indicated a cytolytic clearance of HIV/SIV infected cells as the main protective mechanism for CTLs [49].

Memory T cells have been typically divided into two main subsets based on the expression of the lymph node homing molecules CD62L and CCR7 [50]. Central memory T cells (TCM) express high levels of CD62L and CCR7, whereas effector memory T cells (TEM) express low levels of CD62L and CCR7. However, recent studies demonstrated the existence of a new population of memory T cells designated T memory stem-like cells (TSCM), this T-cell population was first described in mice [51], [52] and more recently a human memory T-cell subset with stem cell–like properties was also identified [53]. This new T-cell population: TSCM have a phenotype similar to those of naïve T-cells (CD44low CD62Lhigh in mice or CD45RO−CD62L+ in humans), but they differ from naïve cells in different characteristics being its vigorous proliferative potential upon restimulation with its specific antigenic peptide, one of the most distinctive [53], [54]. When the distribution of specific memory T-cell populations was compared between both DNA/MVA groups, it was notably revealed that mice immunized with the IL-12_50_+CTB scheme showed significantly higher proportions of CD8 specific T-cells with a central memory phenotype (TCM) (CD44+ CD62L+) (0.1455% vs 0.013%; p<0.02) and with a phenotype compatible with stem cells-like memory T cells (TSCM) (CD44low CD62Lhigh) (1.14% vs 0.098%; p<0.01) (Figure 7A). Thus, T-cell phenotypes that resulted significantly incremented after the DNA-IL-12 plus CTB co-administration were associated with T-cells from earlier stages of differentiation (TSCM and TCM). A similar particular pattern of T-cell memory phenotype distribution as the one found in the present work was already reported for T-cell memory responses in vaccinees after VACV immunizations [55] and also in subjects immunized with other vaccines [31]. Freel and co-workers examined the relationship between CD8 T-cell phenotype and HIV antiviral activity and found that in general vaccinees (with Ad vectors) have HIV-1 specific cells of an earlier differentiation stage (including a high proportion of the phenotype early-memory or “naïve-like”) than controllers, while chronic infected patients have the most-differentiated cells [31]. In relation to previous studies performed in mice in which T-cell memory subpopulations against vaccine antigens were studied, it is striking that in a study published some years ago, it is described that after vaccination of mice with Mycobacterium bovis BCG, the cells expressing a “resting/naïve-like” phenotype (CD44low CD62Lhigh) similar to that found in the present work, were capable of protecting the recipients from a virulent challenge infection [56] . Thus, the finding in the present study in relation to the capacity of IL-12 plus CTB during DNA/MVA intranasal immunizations to augment the proportion of specific T cells with a TSCM-like phenotype is of great importance in the context of vaccine designs, as the recent identification of a human stem cell-like memory T-cell population with increased proliferative capacity and superior protective antitumor responses it was considered of direct relevance to the design of vaccines and T-cell therapies [53].

Collectively, our results demonstrate for the first time that intranasal DNA/MVA immunization schemes can be effectively improved by the co-delivery of DNA-IL-12 plus CTB, inducing elevated HIV-specific CD8 responses at systemic places and more importantly at mucosal sites such as genital tract tissues and ILNs. To denote, these enhanced CTL responses were of superior quality showing higher avidity, polyfunctionality and a broader cytokine profile. Furthermore, by an *in vivo* CTL killing assay we could demonstrate the higher specific CD8 T-cell performance after the IL-12+CTB immunization. Remarkably, the improvements observed were maintained during the memory phase of the adaptive response where we found higher proportions of the specific memory T-cell subpopulations: TCM and TSCM after IL-12 plus CTB nasal co-administration.

## Supporting Information

Figure S1 Evaluation of DNA-IL-12 adjuvant effect after intranasal immunization. Groups of four BALB/c mice were intranasally inoculated as described in the immunization schemes depicted at day zero all mice were DNA primed with the specified DNA vectors described for each group 14 days later all groups were boosted with MVA-EnvB (10^7^ PFU/dose) (A). Ten days after the booster dose, specific cellular immune responses (IFN-γ secreting CD8 T-cells) against Env were quantified by ELISPOT using pooled cells from spleen (B). **: Statistical differences between groups (p<0.01). Differences respect to the control group by one-way ANOVA with Bonferroni's correction post-test.
(TIF)

Figure S2 Specific humoral immune response against gp-120 in mice immunized with mucosal adjuvants. Ten days after the MVA boost, antibody levels against gp-120 were quantified by ELISA in sera (A and B) andvaginal washing (C) samples from immunized mice. (A) specific IgG absorbances, dotted line represents cutt-off for positive responses (B) IgG1/IgG2a absorbance ratios. In A and B, each point represents the mean absorbance values of duplicate determinations of individual sera from four mice per group diluted 1∶50. (C) HIV-1 gp120 specific IgA levels were quantified in vaginal washings of pooled samples from 4 to 6 mice per group diluted 1∶5. Data represent the mean fold increments in the absorbance values of pooled vaginal washings samples of the different experiments, respect to those values detected in pre-immune mice samples. Cut off to consider positive samples were values ≥ to mean values found in naïve samples plus 3SD. *: Statistical differences between groups (p<0.05). NS: Non significant differences respect to the control group by Mann-Whitney test.
(TIF)
