# Supplementary figures and images for: Novel Mucosal DNA-MVA HIV Vaccination in Which DNA-IL-12 Plus Cholera Toxin B Subunit (CTB) Cooperates to Enhance Cellular Systemic and Mucosal Genital Tract Immunity
Source: PLoS One. 2014 Sep 12;9(9):e107524. doi: 10.1371/journal.pone.0107524 (PMC4162600; doi:10.1371/journal.pone.0107524)

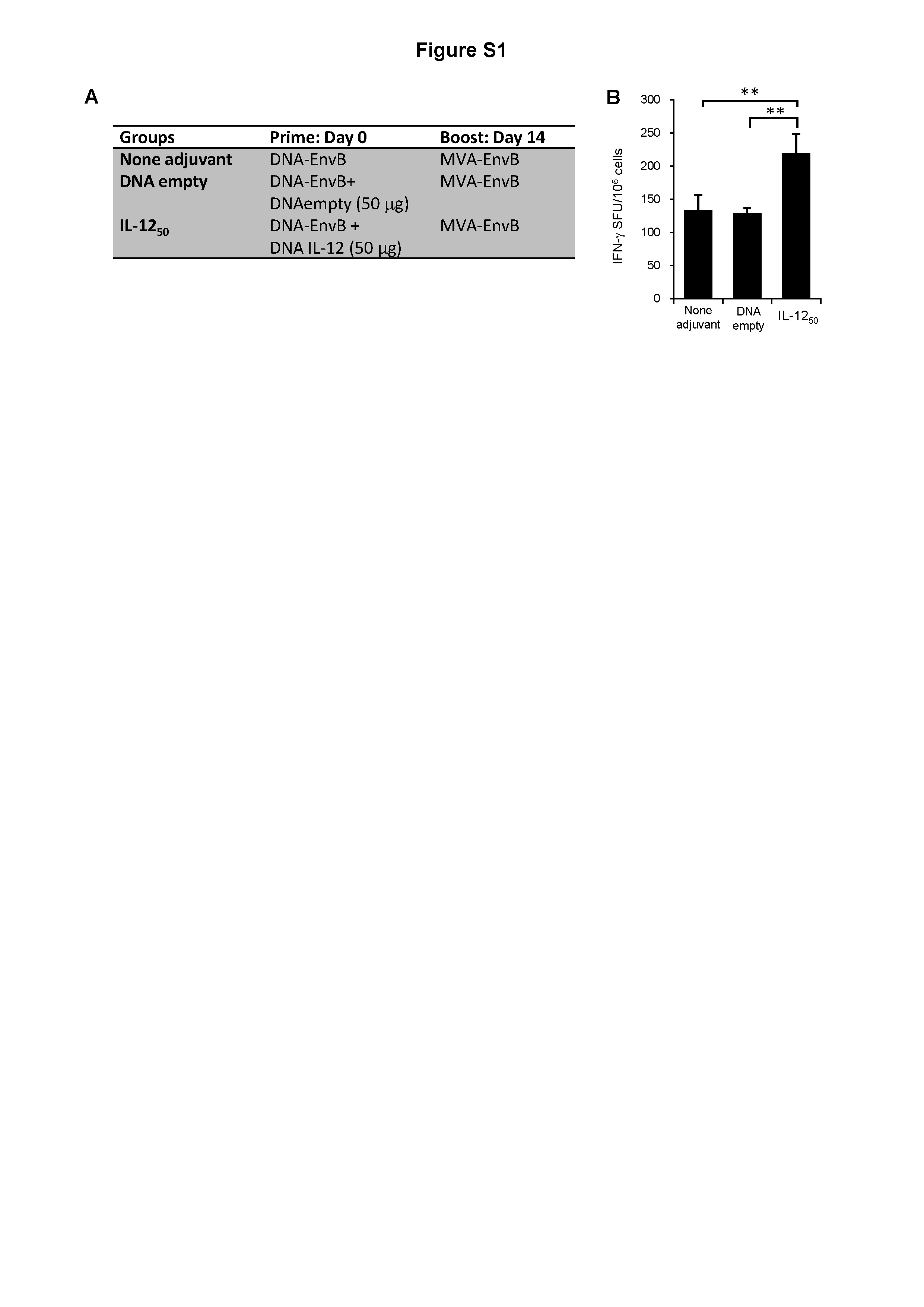

Supplement: Figure S1 — Evaluation of DNA-IL-12 adjuvant effect after intranasal immunization. Groups of four BALB/c mice were intranasally inoculated as described in the immunization schemes depicted at day zero all mice were DNA primed with the specified DNA vectors described for each group 14 days later all groups were boosted with MVA-EnvB (107 PFU/dose) (A). Ten days after the booster dose, specific cellular immune responses (IFN-γ secreting CD8 T-cells) against Env were quantified by ELISPOT using pooled cells from spleen (B). **: Statistical differences between groups (p<0.01). Differences respect to the control group by one-way ANOVA with Bonferroni's correction post-test. (TIF) [file pone.0107524.s001.tif]

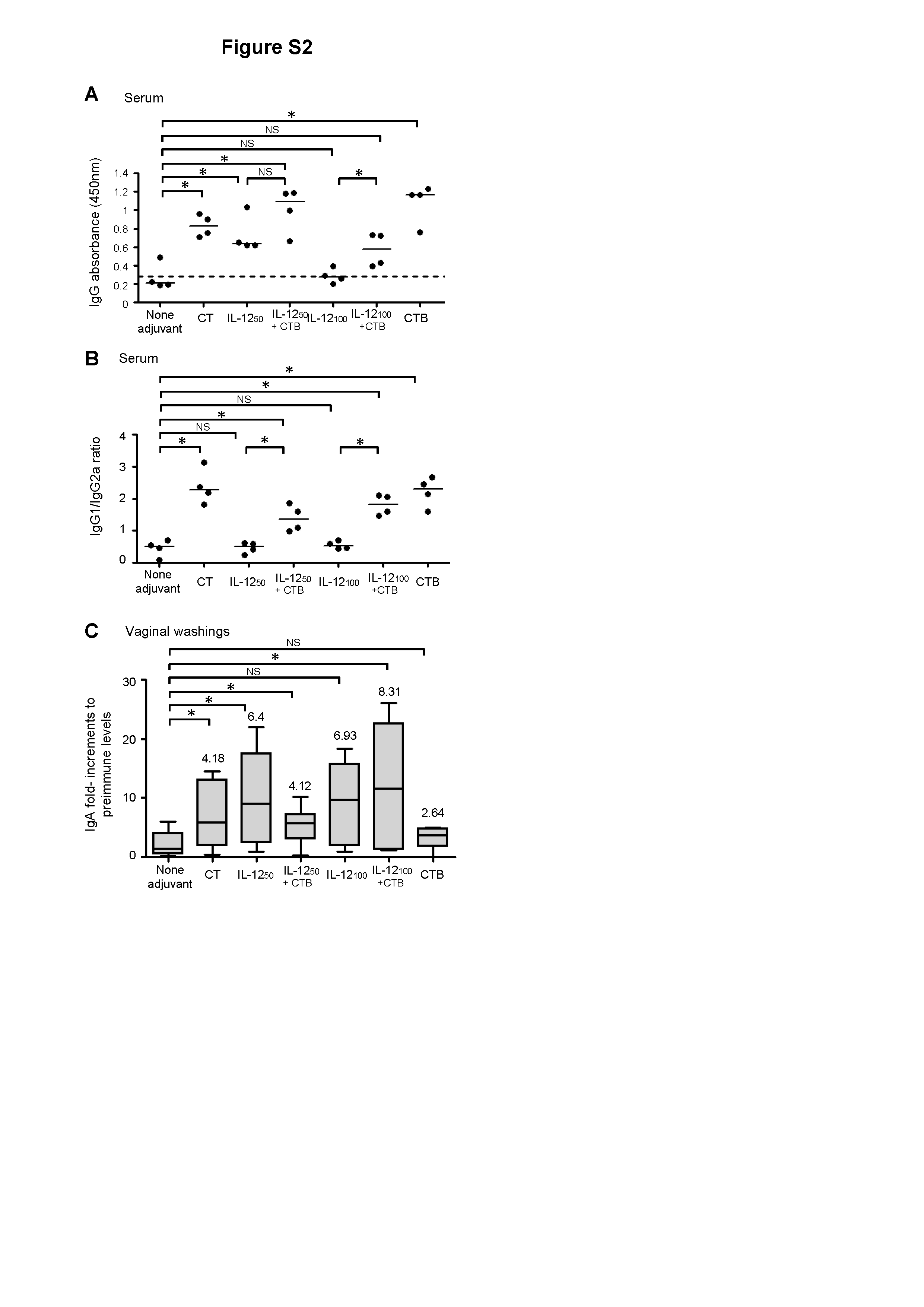

Supplement: Figure S2 — Specific humoral immune response against gp-120 in mice immunized with mucosal adjuvants. Ten days after the MVA boost, antibody levels against gp-120 were quantified by ELISA in sera (A and B) andvaginal washing (C) samples from immunized mice. (A) specific IgG absorbances, dotted line represents cutt-off for positive responses (B) IgG1/IgG2a absorbance ratios. In A and B, each point represents the mean absorbance values of duplicate determinations of individual sera from four mice per group diluted 1∶50. (C) HIV-1 gp120 specific IgA levels were quantified in vaginal washings of pooled samples from 4 to 6 mice per group diluted 1∶5. Data represent the mean fold increments in the absorbance values of pooled vaginal washings samples of the different experiments, respect to those values detected in pre-immune mice samples. Cut off to consider positive samples were values ≥ to mean values found in naïve samples plus 3SD. *: Statistical differences between groups (p<0.05). NS: Non significant differences respect to the control group by Mann-Whitney test. (TIF) [file pone.0107524.s002.tif]
